# Mutational survivorship bias: The case of PNKP

**DOI:** 10.1371/journal.pone.0237682

**Published:** 2020-12-17

**Authors:** Luis Bermúdez-Guzmán, Gabriel Jimenez-Huezo, Andrés Arguedas, Alejandro Leal

**Affiliations:** 1 Section of Genetics and Biotechnology, School of Biology, University de Costa Rica, San Pedro, San José, Costa Rica; 2 School of Statistics, University de Costa Rica, San Pedro, San José, Costa Rica; University of Ferrara, ITALY

## Abstract

The molecular function of a protein relies on its structure. Understanding how variants alter structure and function in multidomain proteins is key to elucidate the generation of a pathological phenotype. However, one may fall into the logical bias of assessing protein damage only based on the variants that are visible (survivorship bias), which can lead to partial conclusions. This is the case of PNKP, an important nuclear and mitochondrial DNA repair enzyme with both kinase and phosphatase function. Most variants in PNKP are confined to the kinase domain, leading to a pathological spectrum of three apparently distinct clinical entities. Since proteins and domains may have a different tolerability to variation, we evaluated whether variants in PNKP are under survivorship bias. Here, we provide the evidence that supports a higher tolerance in the kinase domain even when all variants reported are deleterious. Instead, the phosphatase domain is less tolerant due to its lower variant rates, a higher degree of sequence conservation, lower dN/dS ratios, and the presence of more disease-propensity hotspots. Together, our results support previous experimental evidence that demonstrated that the phosphatase domain is functionally more necessary and relevant for DNA repair, especially in the context of the development of the central nervous system. Finally, we propose the term "Wald’s domain" for future studies analyzing the possible survivorship bias in multidomain proteins.

## Introduction

About 60% of the prokaryotic proteome and 80% of eukaryote proteins are multidomain proteins [1]. It is possibly that proteomes have evolved from a limited repertoire of domain families, where multidomain proteins resulted from combinations of these families [2]. Notably, proteins and domains differ in how well they tolerate variation, suggesting that not all variants are functionally important [3]. Disease-causing variants frequently involve important changes in the physicochemical properties of amino acids, that tend to destabilize proteins and their interactions [4]. These variants occur mostly in the protein core, predominantly in helixes and coil regions, and not so frequently in beta strand structures [5]. Protein-protein interaction regions called interfaces, are also important “hotspots” for disease-causing variants. In fact, when compared to random segregation, disease-causing nonsynonymous single nucleotide variants are preferentially located at protein-protein interfaces rather than surface noninterface regions [6]. Within this interface, variants are preferentially located in the “core” (solvent-inaccessible residues), as opposed to the “rim” (partially solvent-accessible). Interface rim is significantly enriched in polymorphisms, like the remaining non-interacting surface [7]. In addition, variants in ligand-binding sites close to interfaces and residues related to enzymatic function are especially associated with disease [8].

**Fig 1:**
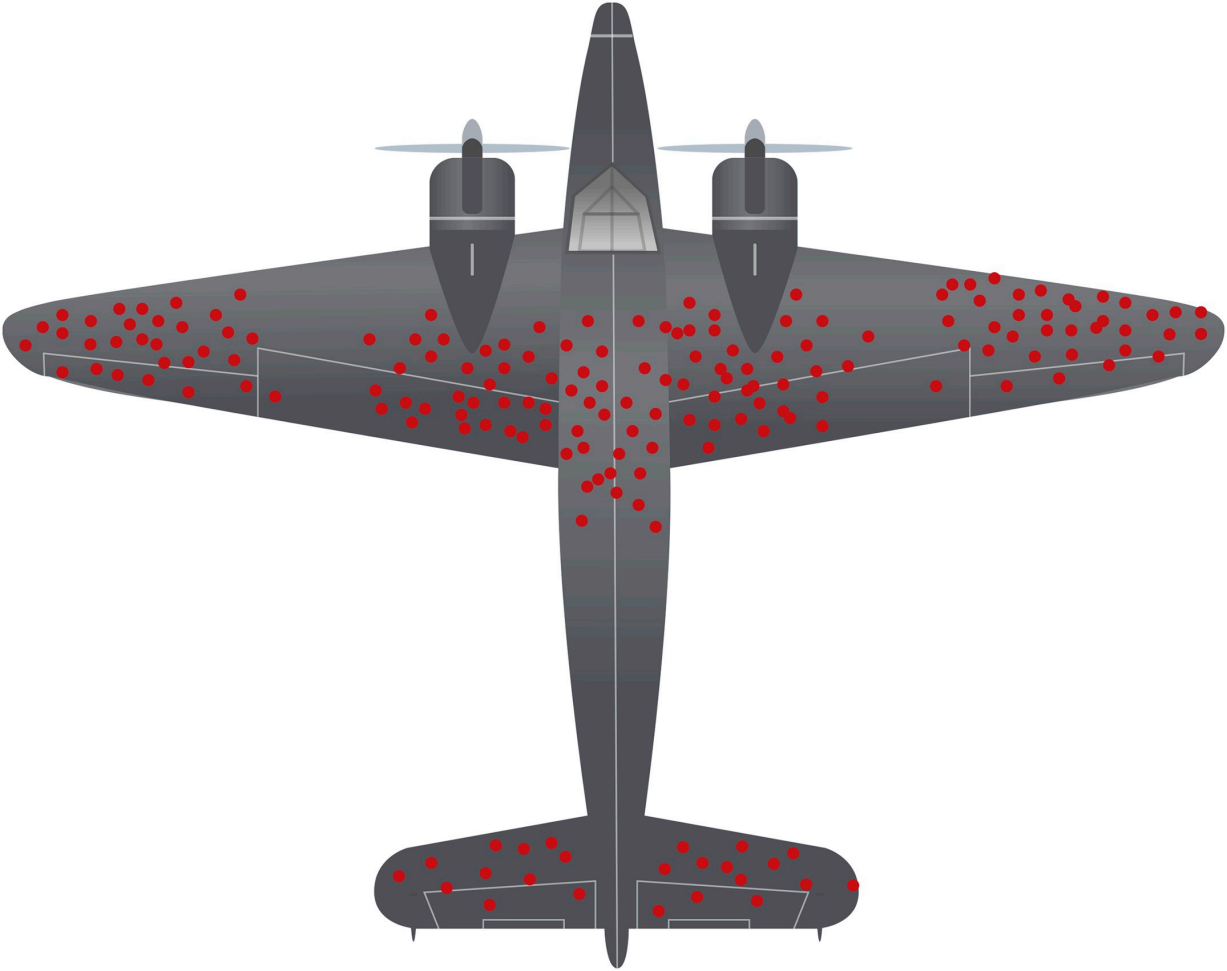
Depiction of Wald’s idea regarding survivorship bias and the overlooking of the events that did not succeed in a selection process. The red dots represent hypothetical areas where an airplane could be damaged and still return.

Most variants we observe in patients are those that manage to be viable despite affecting protein functioning. The variation that results in unviable proteins and eventually unviable organisms, is almost never considered because we hardly see those variants in patients. For instance, it has been shown that proteins and domains vary in their tolerance to non-synonymous point variants according to their mutation rates, degree of sequence conservation and interaction networks [3]. This scenario resembles the one found by the mathematician Abraham Wald during World War II. At that moment, the Center for Naval Analyses (CNA) wanted to determine which sites should be reinforced on their planes based on the damage the planes had once they returned from combat. Wald pointed out that CNA was only considering the planes that had survived their missions for this purpose [9]. Since the planes that had been shot down were not present for the damage assessment, the holes in the planes that returned represented areas where an airplane could be damaged and still safely return (Fig 1). Wald proposed that the Navy reinforce the areas where the returning planes were undamaged since those were the areas that, if damaged, would cause the plane to be lost.

Another interesting case was the one that occurred during World War I, after the introduction of the Brodie helmet and others [10], which was assumed to be the reason for the increased rate of hospitalizations following head injuries. In fact, wearing a helmet did not cause more injuries, but fewer deaths. Soldiers who previously died for head trauma were now surviving so they could be taken to a field hospital. The idea behind both cases was later called survivorship bias, as the logical error of focusing on the events that succeeded a selection process and overlooking those that did not. This concept is not new since different types of biases have been proposed historically in genetics and evolution. One example is the so-called transition bias, as the pattern in which nucleotide transitions are favored several times over transversions, attributed to the fact that transitions are more conservative in their effects on proteins [11].

Analyzing the pathogenicity of variants according to the different domains of a protein can provide significant information about the sites where it is more likely or not to find disease-causing variation [12]. Thus, based on Wald’s observation, we believe that many proteins could be subject to survivorship bias. In other words, we believe that in genotype-phenotype correlation, we are only considering disease-causing variants that are well-tolerated when assessing protein damage. One of these proteins could be the Polynucleotide Kinase 3'-Phosphatase (PNKP), an important nuclear and mitochondrial DNA repair enzyme [13]. This protein consists of 521 amino acids with three characterized domains: an N-terminal FHA (ForkHead-Associated) domain, the phosphatase, and the C-terminal kinase [14]. In Single Strand Breaks (SSBs), PNKP is recruited to the damage site through the interaction between its FHA domain and the X-Ray Repair Cross Complementing one protein (XRCC1) [15]. It can be also recruited by XRCC4 to repair Double Strand Breaks (DSBs) as part of the Non-Homologous End Joining (NHEJ) pathway [16].

Disease-associated variants in PNKP cause a wide pathological clinical spectrum. The most severe phenotype is Microcephaly with early-onset Seizures (MCSZ) [17]. In the middle, Ataxia with Oculomotor Apraxia 4 (AOA4) [18] and in the other end the milder Charcot-Marie-Tooth disease type 2B2 (CMT2B2) [19–21]. The vast majority of variants in PNKP are confined to the kinase domain, both in homozygous but especially compound heterozygous conditions. To date, it is not understood how variants in a single domain can cause such a broad clinical spectrum, so it is speculated that the kinase domain is the one that determines the degree of pathogenicity. In this paper, we analyze the variants observed in each domain of PNKP in order to determine whether they are subject to mutational survivorship bias or not. To do this, we combine *in silico* analysis, published experimental data and open sequencing data to integrate both structural and functional features.

## Results

### Multiple sequence alignment

**Fig 2:**
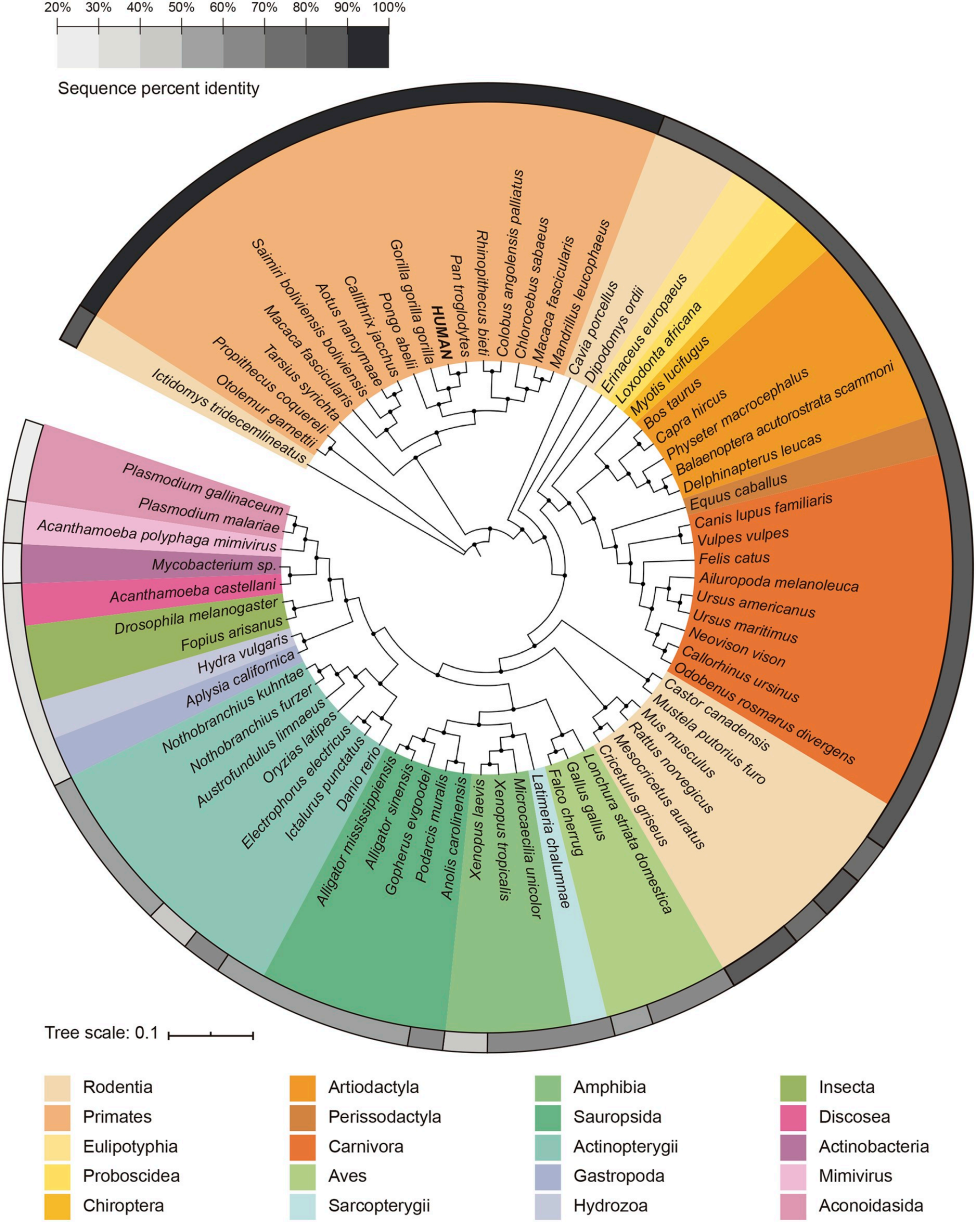
Phylogenetic tree of the seventy species used in the multiple sequence alignment. Species are colored according to their taxonomic group. The gray circular scale represents the percentage identity with respect to the human protein sequence of PNKP.

First, we wanted to analyze some general features of PNKP. From all the available protein sequences in the Uniprot database, seventy sequences of twenty taxonomic groups were selected. We performed a Multiple Sequence Alignment using Fast Fourier Transform (MAFFT) against the human PNKP sequence (see Methods for the ID number of each sequence). We generated a phylogenetic tree based on this alignment with the ITOL platform [22]. We also ran a BlastP analysis to determine the percentage identity among species and we noticed that human PNKP has a low percentage of sequence identity compared to some close groups (Fig 2).

As expected, the group of primates was the closest to the human, with identities between 90–100%. The species belonging to the other eight orders of mammals presented a range between 80–90% (e.g. *Mus musculus*: 80.88%). The species within the group of birds, reptiles, and amphibians had a range between 50–70% (e.g. *Gallus gallus*: 66.19%). Different species of fish showed even a lower range (50–60%). The rest of the species had percentage identities below 50% (*e*.*g*. Drosophila melanogaster: 40.16%).

### Percentage identity analysis of SSBR proteins

To further confirm the low percentage identity between the human sequence of PNKP and other species, we compared the degree of identity between the human sequences of different enzymes from the Single-Strand Break Repair (SSBR) pathway with respect to ten vertebrate species. Protein sequences of PNKP, PARP1, XRCC1, POLB, and LIG3 were obtained from Uniprot and analyzed using the BlastP tool [23]. We identify the percentage identity of *Pan troglodytes*, *Equus caballus*, *Sus scrofa*, *Canis lupus*, *Felis catus*, *Mus musculus*, *Rattus norvegicus*, *Gallus gallus*, *Xenopus laevis*, and *Danio rerio* with respect to the corresponding human sequence of each protein (see Methods for the ID number of each sequence).

**Fig 3:**
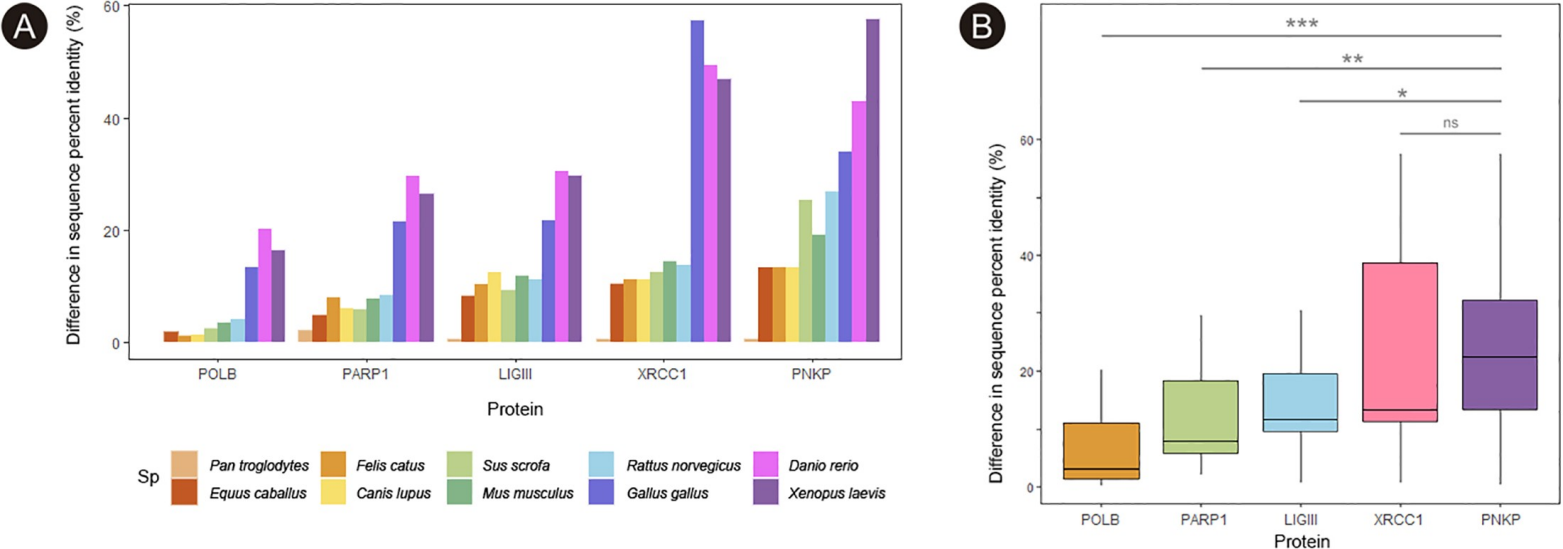
Percentage identity analysis of SSBR proteins. A) Comparison of the difference in the percentage identity between the human sequence of each SSBR protein and several species. The higher the bar, the more different is the sequence of each organism with respect to the human. B) Comparison of different SSBR proteins according to the variability in the sequence percentage identity between species (* p<0.05; ** p<0,01; *** p<0,001; ns: not significant).

We later plotted the percentages corresponding to the differences in the sequence identity between species. The protein sequence that showed the lowest difference between species was POLB, followed by PARP1, LIGIII, XRCC1 and finally PNKP (Fig 3A). Surprisingly, the difference between the human PNKP sequence and *Mus musculus* (mouse) was almost 20%. To further evaluate this difference, an ANOVA test was performed and we confirmed that there is a smaller gradient, and thus less differences between species, for POLB protein sequence, and a larger gradient, with a notable difference between species for PNKP and XRCC1 (F value = 13.368; p-value = 8.9 x10-7; Fig 3B).

Using Tukey’s intervals for multiple comparisons, we observed that POLB presented lower differences among species than PNKP and XRCC1 (p < 0.05), while no differences were found with respect to PARP1 and LIGIII (p > 0.05). No significant differences were found between PARP1, LIGIII and XRCC1 (p > 0.05). PNKP shows bigger differences in sequence identity than POLB (p = 0.0000035), PARP1 (p = 0,001) and LIG III (p = 0.013). No difference was found between PNKP and XRCC1 (p = 0,97).

### Conservation analysis

**Fig 4:**
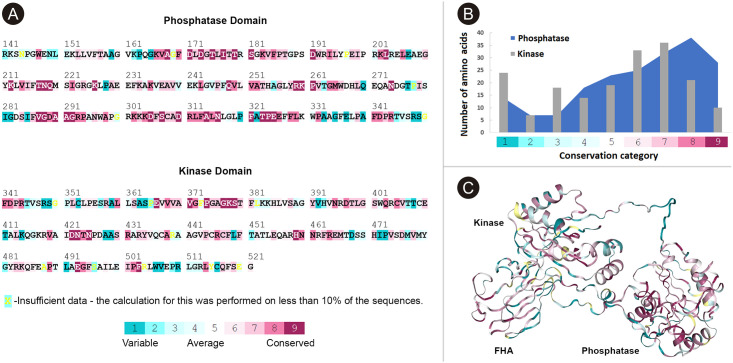
Conservation analysis of the phosphatase and kinase domains. A) Color code according to the degree of sequence conservation for each domain. Conservation scores are calculated based on the previous MSA. B) Number of amino acids classified according to their conservation scores for each domain. C) Prediction of the 3D structure of human PNKP colored according to the degree of conservation for each domain. The linker between the FHA and phosphatase domain shows low conservation scores, while the degree of greater conservation is in the phosphatase domain.

The evident differences that we found between proteins from the same DNA repair pathway, made us wonder if the degree of sequence conservation of PNKP was different between domains. To analyze this, the previous multiple sequence alignment (MSA) was used as input for the ConSurf server to get and compare the conservation scores of the amino acids within the phosphatase (from amino acid 146 to 337) and kinase domain (from 341 to 521) (Fig 4A). There was a total of 192 observations for the phosphatase domain and 181 for the kinase, corresponding to each amino acid. This makes the results using absolute numbers almost equal to those using relative numbers. Homogeneity chi-squared test showed that both domains have different conservation scores (χ^2^ = 22.865, df = 8, p = 0.0035; Fig 4B), with higher degree of conservation in the phosphatase domain. Specifically, in the cases of conservation scores 8 and 9, the percentage of observations from phosphatase domain was 34.4%, while, for kinase this percentage was only 16.99%. On the other hand, for lower conservation scores (from 1 to 3), the percentage in the phosphatase domain was 14.59%, while for the kinase was 24.94%. ConSurf also predicted the 3D structure of PNKP using Modeller and HHPred Model algorithms to color the structure according to the conservation scores (Fig 4C). Interestingly, the linker between the FHA and the phosphatase domain has very low conservation scores. This may also account for the differences observed between species.

### Disease-propensity hotspots in PNKP

Once we determined that the phosphatase domain is more conserved and generated the best 3D structure of PNKP, we used the SuSPect server [24] to predict the disease-propensity scores (DPS) (from 0–100) for each amino acid, following saturation mutagenesis of the entire protein. Notably, SuSPect currently outperforms several common servers used to predict variant pathogenicity and disease-susceptibility-based single amino acid variant phenotype prediction. The improved performance of SuSPect is derived from the integration of several features, specially the degree of centrality in the protein-protein interaction network. Other factors include the position-specific scoring matrix, the difference between Pfam hidden Markov model (HMM) emission probabilities for the wild type and mutant amino acids at any position, Jensen-Shannon divergence, among other features. As input for this server, we used the structure generated in Modeller and the FASTA sequence of PNKP.

**Fig 5:**
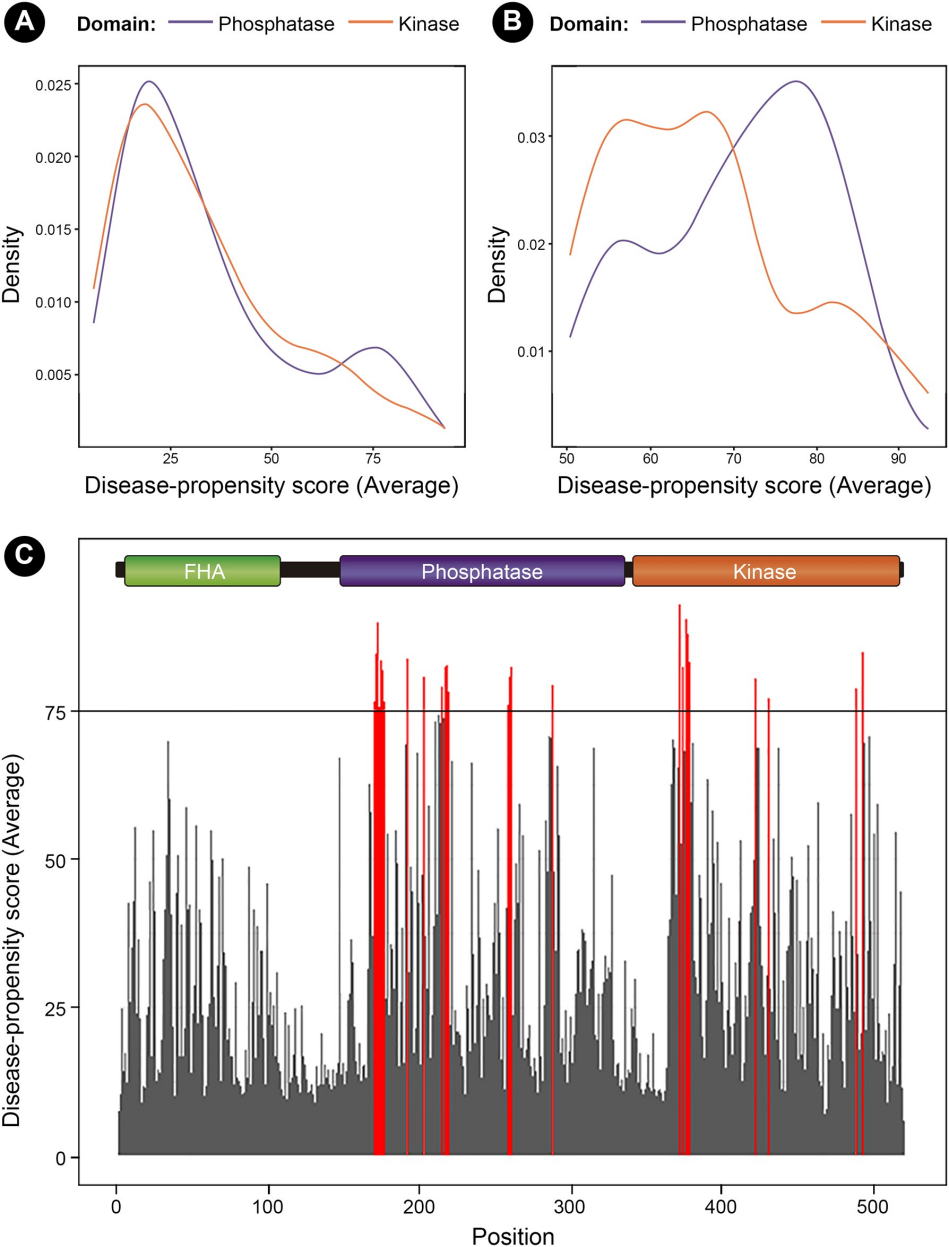
Disease-propensity plots of PNKP. A) Distribution of the average Disease-Propensity scores for the amino acids within the phosphatase and kinase domain of PNKP. Notice that there is a higher number of residues showing DPS > 75 in the phosphatase domain. B) The differences between average DPS is more evident when only DPS > 50 are considered. C) DPS for the entire protein, showing several hotspots in the phosphatase domain. All regions with an average beyond the critical value of 75 are colored in red, but only consecutive positions with averages over the critical value are considered disease-propensity hotspots.

Once we generated the DPS for the entire protein, the average score of the 19 possible variants for each amino acid was calculated, and the distribution of said averages was plotted. As we noticed that values for the phosphatase and kinase domain presented an asymmetric distribution (Fig 5A), we next plotted only the DPS greater than 50 (Fig 5B). We performed the Kolmogorov–Smirnov test to verify the differences of both distributions and the result was almost statistically significant (D = 0.30438, p-value = 0.052). Then, we plotted the average DPS for every amino acid, defining the critical value as the 95^th^ percentile, close to a DPS of 75 (Fig 5C). This means that, for a position to be considered as part of a “disease-propensity hotspot”, the region must present an average higher than 75 for all possible single amino acid variants. The only consecutive positions with averages over the critical value were from 170 to 177, from 217 to 219, and from 259 to 261 in the phosphatase domain. In the kinase domain only amino acids from 377 to 379 exceeded the threshold. Since this is an average, individual values could be much higher for some of the said positions.

### Human PNKP 3D structure prediction

Once we characterized the disease-propensity hotspots of PNKP, we wanted to combine this information with some structural analyses. For this purpose, since there is no crystal structure for the human PNKP in the RCSB Protein Data Bank, different servers were used to perform homology modeling. Fortunately, the crystal structure for the murine Phosphatase-Kinase domains is available (PDB ID: **3ZVL**) [25], which results very convenient for the homology modeling approach. Swiss-Model generated the best model using only two templates (3ZVM.1.A, 1YJM), but it was not able to generate the complete structure as it did not find a template to model the linker between the FHA and phosphatase domains, generating a gap in the 3D model. Interestingly, this is the same region with a very low conservation score from Fig 4C. For this reason, this model was discarded. Since ConSurf generated its own model using the Modeller server, we included this model to be evaluated alongside the others. Modeller used six different templates (**3ZVL_A**, 1UJX_A, 1YJ5_C, 2BRF_A, 3KT9_A, 1LY1_A), Phyre2 selected 7 templates (D2BRFA1, D1UJXA, C1YJ5B, C3ZVMA, D1YJMA1, D1QU5A, C1YJ5C) while I-TASSER used ten templates to model the structure (**3ZVLA**, 1YJ5B, 1UJXA, 2BRFA, 1YJ5B, 1YJ5, 1YJMA, 1YJ5, 1YJ5B, 3KT9A). From all the models generated with I-TASSER, we chose the model with the lowest C-score (-2.22).

The Ramachandran plots of each model were generated with the Swiss-Model Structure Assessment tool. According to these plots, the model generated by Modeller presented the highest number of amino acids in a favored position and the lowest number of outliers (S1 Fig). For this reason, we chose this model for further analysis. More detailed results of all the rubrics evaluated in MolProbity for each model are available in Supporting information (S1 Table). In addition, we performed a structural alignment between our 3D structure generated with Modeller and the mouse Phosphatase-Kinase crystal structure of PNKP (PDB ID: 3ZVL). Notably, both structures were almost perfectly aligned (TM-score: 0.99357; RMSD: 0.25 Å).

### The PNKP ligand-binding sites prediction

**Fig 6:**
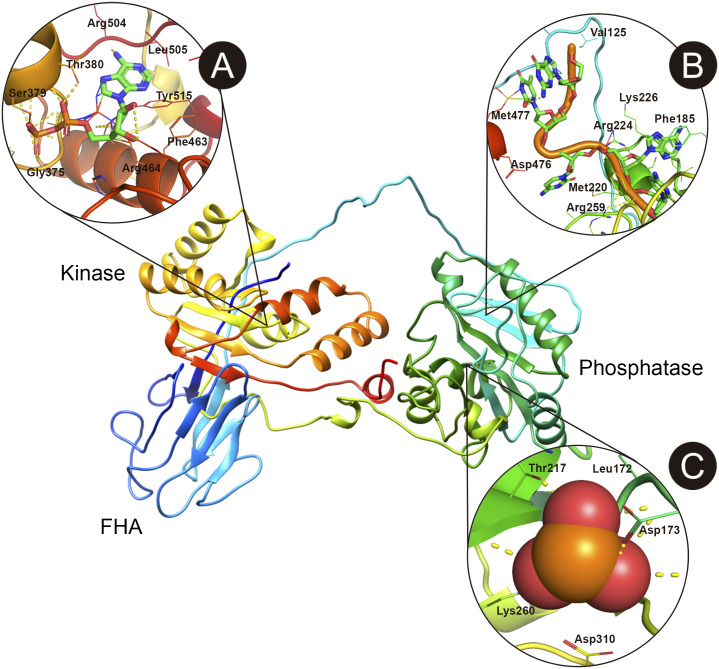
Structure of human PNKP showing the most important ligand-binding sites predicted *in silico*. A) Pocket showing the ATP/ADP binding site in the kinase domain. B) DNA binding site in the phosphatase domain. C) Phosphatase active site. Most predictions coincide with previously experimental results. Figure was elaborated with PyMol 2.3.

Once the chose the best structure, we wanted to analyze if predicted disease-propensity hotspots match the functional ligand-binding sites of PNKP. As these sites have been already determined at the experimental level, we wanted to determine if we were able to get comparable results *in silico*. For this purpose, we used the 3D structured predicted with Modeller as input for the COACH [26] and COFACTOR [27] servers. From all the predictions, we only chose the residues with the highest C-score (confidence score of the prediction from 0 to 1, where a higher score indicates a more reliable prediction). The highest C-Score for the DNA-binding site was given by COFACTOR (0.61) for the following residues: D171, N218, Q219, M220, R259 and F306 (Fig 6). COACH predictions presented a slightly lower C-Score (0.57) for the same residues but also included the following: V184, F185, S221, R224, K226, A255, M477. Some of these residues coincide with the hotspots we found from amino acids 170–177 and 259–261 (Fig 5). Only COACH predicted the ATP/ADP-binding site in the kinase domain, with a C-Score of 0.15 and the following residues: G375, A376, G377, K378, S379, T380, N460, F463, R464, F503, R504, L505, W506, Y515 (Fig 6). This site also coincides with the kinase disease-propensity hotspot from 377–379. Most of these predictions coincide with experimental results [25, 28].

For the phosphate group binding site (phosphatase active site), COFACTOR predicted with greater C-Score (0.42) the residues L172, D173, T217, N218, K260 (Fig 6C). These residues also match with the disease-propensity hotspots we found in the phosphatase domain from amino acids 170–177 and 259–261. COACH also predicted three amino acids for the Mg^+2^ binding sites (D171, D173, D289) which also matched a hotspot within the phosphatase domain (C-Score: 0.20). This is also in agreement with experimental results [25, 28]. Although we could not predict the kinase DNA-binding site, it was experimentally demonstrated that the DNA backbone approaches the kinase domain near a positively charged surface including R403, R482 and R483 residues [28]. Interestingly, these sites presented low DPS on average (R403 = 14.85; R482 = 39.35; R483 = 25.8).

To add one more layer of complexity, we also analyzed the potential post-translational modifications (PTM) sites of PNKP. NetPhos 3.1 predicted S47, S114, T118, S126, S143, S221, S280, S307, T323, S347, T407 as possible phosphorylation sites (prediction score > 0.90). To confirm this prediction, we reviewed the Phospho.ELM and PhosphoSitePlus v6.5.9.1 database, which contain *in vivo* and *in vitro* phosphorylation data. Phospho.ELM confirmed the phosphorylation of S114, T118, T122, S126. PhosphoSitePlus also confirm these sites but also reported S12, T109, T111, T122, N144, G188, S364 and S379, although some with few references. Other PTMs were reported, as acetylation in K226, K233 and K242 and ubiquitination in K142, E151, K183, K226, and K484. However, these sites do not coincide with disease-propensity hotspots nor mutation sites.

### Structural damage of PNKP variants

**Fig 7:**
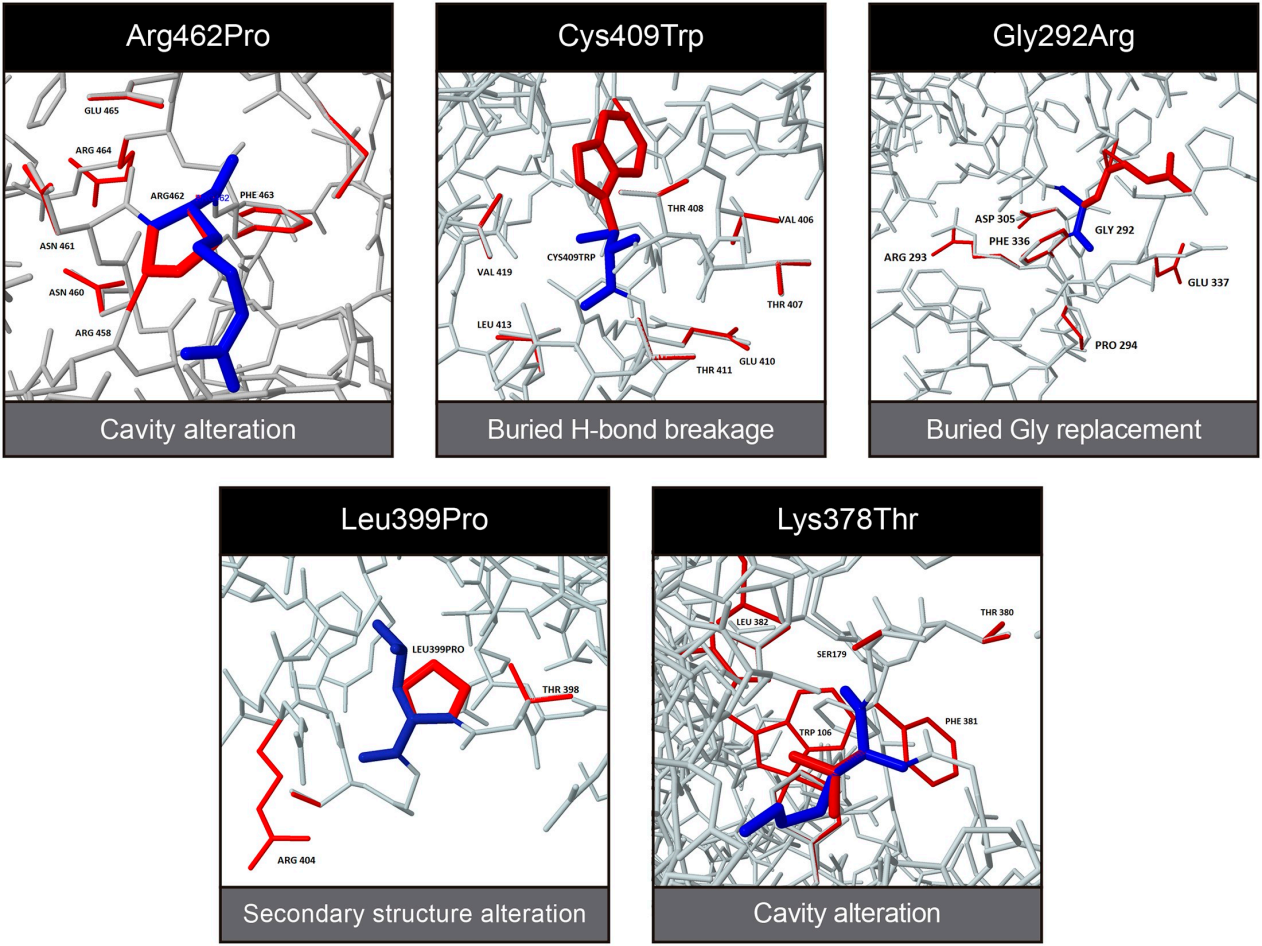
Missense3D prediction of structural changes generated by Single Amino acid Variants reported in patients with PNKP-associated diseases. Wild-Type residues are represented in blue while the mutant amino acids and the nearby residues affected by them are represented in red.

As we found that the disease-propensity hotspots matched some predicted ligand binding sites, we next evaluated whether Single Amino acid Variants reported so far in patients also coincided with these hotspots. We also evaluated the structural damage of each variant using the Missense3D server. Dynamut were also used to analyze the impact of mutations on protein dynamics and stability resulting from vibrational entropy changes. We included the following missense variants [19]: Arg462Pro, Lys378Thr, Gly375Trp, Leu399Pro, Cys409Trp, Pro343Leu, Gly442Ser, Leu176Phe, Gly292Arg and Glu326Lys (Fig 7). Notably, most of these residues are located within the core of the protein.

#### Kinase domain

Although Arg462Pro is not located within a hotspot site, according to the Missense3D prediction, the variant generates an alteration that affects nearby amino acids (460,463,464) leading to the expansion of cavity volume by 117.72 Å^3. These nearby amino acids are part of the ATP/ADP binding sites in the kinase domain. In addition, the change in folding free energy (ΔΔG) is predicted to be stabilizing. Lys378Thr is located within a hotspot associated with ATP/ADP binding site. Missense3D predicted a contraction of cavity volume by 74.736 Å^3 and the disruption of all sidechain/main-chain H-bond (s) formed by this buried Lys residue (RSA 3.4%). The variant is predicted to be destabilizing, increasing protein flexibility. Gly375Trp is also within a hotspot (ATP/ADP-binding site). However, it does not generate any structural alteration according to Missense3D and the change in ΔΔG is predicted to be stabilizing. The rest of the variants in the kinase domain do not present any clear tendency (Table 1). Pro343Leu and Gly442Ser show low DPS and intermediate conservation score. Neither generate structural alterations. Although the ΔΔG is predicted stabilizing for both Pro343Leu and Gly442Ser, the latter is associated with an increase in molecule flexibility. Leu399Pro changes ‘H’ (4-turn helix) to ‘T’ (hydrogen-bonded turn). Cys409Trp generates a structural alteration that disrupts all sidechain/main-chain H-bond (s) formed by this buried CYS residue (RSA 2.2%) and the ΔΔG is predicted to be stabilizing, decreasing molecule flexibility.

**Table 1:** Structural analysis of missense variants reported in the kinase and phosphatase domains of PNKP.

Variant	Conservation Score (1–9)	DPS (0–100)	Hotspot	Binding Site	ΔΔG (Kcal/mol)	Structural Damage
p.Arg462Pro	6	23	No	Nearby	0.201	Cavity expansion
p.Lys378Thr	9	92	Yes	Yes	-0.957	Cavity expansion
p.Glys375Trp	6	86	Yes	Yes	1.270	No
p.Pro343Leu	7	36	No	No	1.543	No
p.Gly442Ser	5	9	No	No	-1.822	No
p.Leu399Pro	7	64	No	No	-1.661	Secondary Structure
p.Cys409Trp	3	71	No	No	0.059	H-Bond breakage
p.Leu176Phe	7	87	Yes	Yes	0.188	No
p.Gly292Arg	8	37	No	No	1.063	Buried Gly replacement
p.Glu326Lys	8	28	No	No	-0.788	No

#### Phosphatase domain

Only three disease-associated variants have been reported in the phosphatase domain so far [17, 29]. Leu176Phe is located within a disease-propensity hotspot (DNA-binding site) but it does not generate structural damage and the ΔΔG is predicted to be stabilizing. Gly292Arg replaces a buried GLY residue (RSA 5.9%) with an exposed ARG residue (RSA 10.4%). Finally, Glu326Lys does not generate structural damage nor is it a ligand-binding site but the ΔΔG is predicted destabilizing, decreasing protein flexibility.

### PNKP variants in the general population

We also wanted to analyze variants segregating in the population, so we looked for PNKP variants reported in the gnomAD v2.1.1 dataset, which includes more than 141.000 individuals. We found a total of 424 missense variants, but only 193 in the kinase domain, 132 in the phosphatase and 71 in the FHA domain. Based on these results, observed mutations in the kinase domain are higher than expected compared to the phosphatase domain (d.f. = 1, χ^2^ = 11.449; p < 0.001). In addition, we found 12 *nonsense* variants in the kinase and only 3 in the phosphatase domain. From the all the frameshifts reported (31), we observed 23 in the kinase and only 8 in the phosphatase. Of all missense variants, only 12 were reported in homozygous condition, but they represented a very small portion since each variant has a high allelic count.

Interestingly, there are two variants with a high number of homozygotes exclusive to Asia and others exclusive to Africa (S2 Fig). Of the 12 homozygous mutations found, 5 were in the phosphatase domain, but only one (Arg301Trp) is predicted to generate a structural alteration. For this variant, Missense3D predicted a cavity alteration, but this amino acid is not located within any hotspot and the variant has a low DPS (31) and low conservation score (3). In general, the other variants have low DPS, as well as low conservation scores respectively: Tyr196Asn (16; 6), Arg139His (11; 3), Arg180Ser (18; 5), Glu508Lys (18; 5), Arg462Gln (13; 6), Ala441Gly (44; 6), Arg301Trp (31; 3), Thr217Ser (39; 9), Arg141Gln (11; 6), Gly244Arg (12; 4). Notably, most of the residues (except Thr217 and Ar462) are located on the protein’s surface.

### Variant tolerability analysis

**Fig 8:**
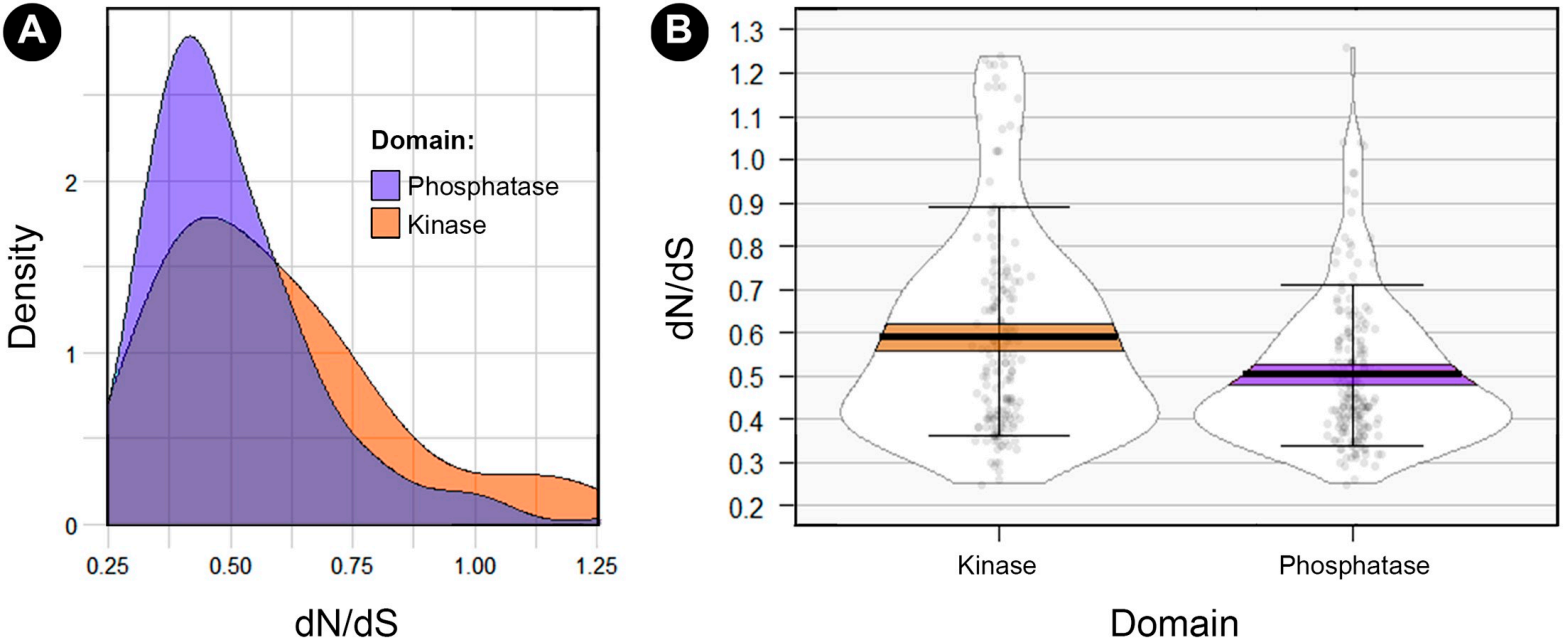
Analysis of the dN/dS ratio between the phosphatase and kinase domain. A) Comparison of the distributions of dN/dS values for each domain showing higher density of low dN/dS values for the phosphatase domain (D = 0.191, p-value = 0.002). B) Comparison of the mean dN/dS values between domains, showing a higher tolerability in the kinase domain (W = 21063, p-value = 0.0004).

**Fig 9:**
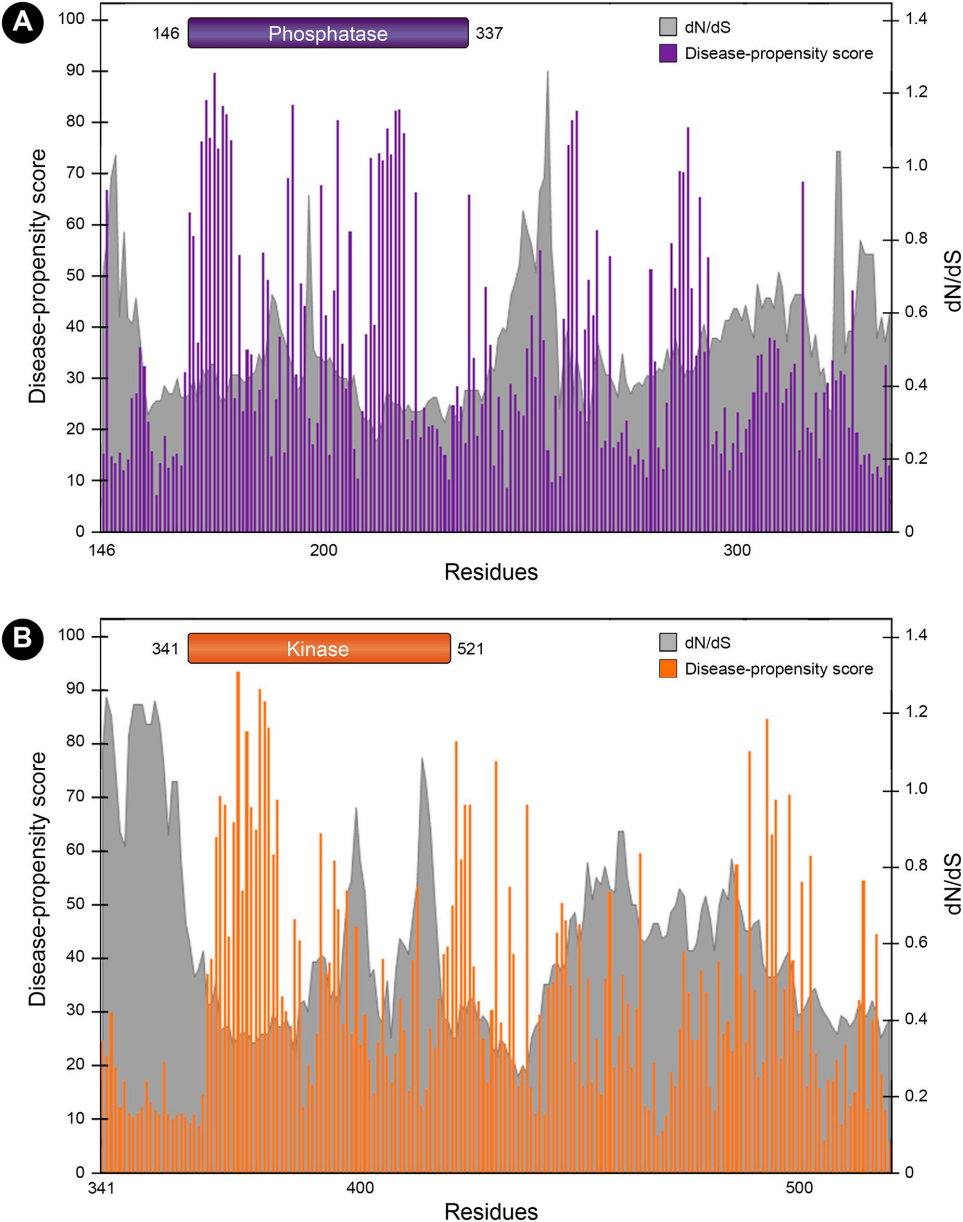
Mutational tolerance landscape in the phosphatase and kinase domain. The gray shaded areas represent the dN/dS values for each position and the corresponding mutational tolerability (the lower the ratio, the lower the tolerance). Bars represent DPS (0–100), so that "valleys" are regions with the lowest dN/dS ratios (highly intolerant to variation) and the highest DPS.

To obtain further information on the tolerability of PNKP to variation, we used the MetaDome server to compare the mutational tolerance landscape of each domain in terms of the nonsynonymous over synonymous ratio (dN/dS). This is calculated based on the single nucleotide missense and synonymous variants (SNVs) from gnomAD in a protein-coding region. This score is later corrected for the sequence composition of the protein coding region based on the total possible missense and synonymous SNVs. When we compared the density distribution of each domain, we found a higher density of low dN/dS values in the phosphatase domain (D = 0.191, p-value = 0.002; Fig 8A). The same was found when we compared the mean dN/dS values between domains (W = 21063, p-value = 0.0004; Fig 8B). It was not surprising that when we analyzed both disease-propensity scores and this tolerance landscape plot, there was an evident overlap. For those “valleys” in the landscape (low dN/dS values relative to intolerant regions) the DPS was higher, compared to the “hills” that presented the lowest DPS (Fig 9).

## Discussion

### PNKP evolutionary and structural background

Current bioinformatics tools provide useful *in silico* results that combine theoretical-experimental knowledge to better understand the relationship between protein structure, function, and disease. In our analysis, multiple sequence alignment was very informative to determine that human the PNKP protein sequence has a low identity among the species analyzed. As mammals represent more than 50% of the species analyzed, it would be expected that the percentage identity would higher for a DNA repair enzyme with a function as important as that of PNKP. Surprisingly, we found a large difference between the percentage identity of PNKP with respect to POLB, PARP1, and LIGIII among species. As we noticed the low conservation in the linker between the FHA and phosphatase domain, we assume that part of this difference could be explained from the low conservation of this region (~40 amino acids).

After the sequence identity analysis, we performed the sequence conservation analysis and we found that as expected, the phosphatase domain was more conserved give the higher number of amino acids with higher conservation scores and less with very low conservation scores. Therefore, we believe that the evolutionary rate of the kinase domain in PNKP is higher, which is supported by the observed higher mutational tolerability. Curiously, it has been reported that kinase proteins derived from a single protein structural fold compared to protein phosphatases, that are associated with many different protein folds and catalytic mechanisms [30]. More research is needed to elucidate the reason behind the differences we observed in terms of conservation, but we believe that this could be related to the functional importance of the phosphatase domain in PNKP especially during development.

Although the crystal structure of human PNKP has not been determined, we obtained very good results when using different servers to predict it by homology modeling. In fact, the structural alignment showed that our model was comparable (very low RMSD and high TM-Score) to the murine crystal phosphatase-kinase structure. The accuracy of our model was very useful to predicted protein ligand binding sites, which was confirmed by comparing our predictions with previous experimental results. Likewise, we were able to confirm that three-dimensional protein models are effective to infer the pathogenic effect of a variant.

### Variant pathogenicity analysis

The three pathogenic variants reported in the phosphatase domain are associated with the most severe phenotype (MCSZ), however, none are predicted to generate a significant structural alteration. As expected, Gly292Arg and Glu326Lys presented low DPS but high conservation scores. Gly292Arg has only been reported as compound heterozygous together with Ala55Ser. The latter is a variant located in the FHA domain that also has low DPS (= 39) but high conservation score (= 8) and without predicted structural damage. The Leu176Phe variant is located within a disease-propensity hotspot and was reported to exhibit reduced levels of both DNA phosphatase and DNA kinase activity at 30°C [31]. One may think that this mutation should not be viable, especially in a hotspot site, but it has only been reported in a heterozygous condition, together with Thr424GlyfsX48. As the latter variant only affects the kinase activity, we assume that it compensates for the affectation generated by Leu176Phe. In fact, it was demonstrated that T424Gfs48X exhibited levels of DNA 3’-phosphatase activity comparable to WT despite having little DNA 5′-kinase activity [31]. Interestingly, the homozygous T424GfsX48 allele (causing MCSZ in humans) was lethal in mice at the embryonical level [32]. Thus, we believe that Leu176Phe mutation as others in PNKP can be under some degree of interallelic complementation as this is most often observed in multi-domain proteins [33]. Notably, Glu326Lys has only been reported in homozygous condition in severely affected individuals [17]. According to this, one may think that this mutation also represents an exception for our proposal, but it is more a confirmation since it was demonstrated that E326K exhibited almost normal levels of both DNA 3′-phosphatase and DNA 5′-kinase activity at 30°C [31]. However, this variant results in a 10 to 20-fold reduction of PNKP cellular levels. In line with this, we found that this mutation is predicted to be destabilizing (ΔΔG = -0.788 kcal/mol). This could be related to the impaired interaction reported for this variant between PNKP and the XRCC4-LigIV complex [16]. Thus, the severity of the phenotype generated by this mutation further confirms that insults in the phosphatase domain (especially in homozygous condition) are highly intolerant.

In the kinase domain, the Lys378Thr mutation affects the ligand-binding site and is predicted very pathogenic, only reported in MCSZ. Gly375Trp is located also in the ligand binding site (ATP/ADP) and although it does not generate any structural alteration, it is predicted pathogenic as well. However, Gly375Trp has only been associated with AOA. In this case, as both can be in homozygous condition, the genotype, and the prediction of its effect on protein activity is not related to the observed phenotype. To support this, Arg462Pro also affects nearby residues of the ATP/ADP binding site in the kinase domain but can cause MCSZ when homozygous or AOA when accompanied by Leu399Pro. Thus, variants in the active site of the kinase domain can not only be tolerated but can be found in a homozygous condition. Likewise, Leu399Pro, Cys409Trp and Pro343Leu (all in the kinase domain) usually occur in a compound heterozygous condition, accompanied by a second allele with more serious insults in the kinase domain, usually deletions or frame shift variants [19].

From all the missense variants reported in gnomAD, a higher number were located in the kinase domain. None of the homozygous variants reported in the phosphatase domain are within any hotspot. In addition, the general prediction for these variants is benign, possibly because most of them are located at the surface of the protein. This is very important since surface residues are less evolutionarily conserved and do not present a restricted conformational space, leading to potentially more viable physicochemical changes [7].

### Mutational survivorship bias

Most of the disease-causing variants in PNKP are confined to the kinase domain. However, not all combinations are equally pathogenic as many patients harboring only kinase mutations can present either a severe (MCSZ) and/or milder phenotype (AOA4/CMT). In contrast, only three pathogenic variants have been reported in the phosphatase domain and the three of them are associated to the most severe phenotype (MCSZ). A few novel variants in the FHA domain are associated with both AOA4 and MCSZ [34, 35]. Although the FHA domain was not considered in our work, we do not rule out the possibility of the possibility that it has a trend comparable to the phosphatase domain. Remarkably, most pathogenic variants in PNKP are presented as compound heterozygotes with only few reported as recessive. This suggests that there is a gene dose effect on the pathogenicity of PNKP variants as for many enzymes [36, 37]. In fact, it has been determined that deleterious variants are more likely to be recessive than less deleterious mutations [38]. Some variants in PNKP may be subject to interallelic complementation. In fact, E326K is the only recessive mutation in the phosphatase domain, but it has been shown to affect neither phosphatase nor kinase activity, but to decrease cellular levels of PNKP. The other mutations in phosphatase are accompanied by another mutation in the kinase that does not affect the phosphatase activity of the protein.

At the functional level, PNKP kinase and phosphatase activities do not act in concert on substrates containing both 5'-hydroxyl and 3'-phosphate termini, but PNKP phosphatase activity is much faster than the kinase activity. In addition, the 5'-phosphorylation of strand breaks containing a 3'-phosphate and 5'-hydroxyl depends on the preprocessing by the phosphatase activity of PNKP [39]. Likewise, it was demonstrated that the overexpression of a wild-type recombinant PNKP, but not 3′-phosphatase-dead PNKP, can override the requirement for PNKP interaction with XRCC1 for rapid rates of SSBR following oxidative stress [40]. This can be related to the fact that PNKP preferentially binds to 3'-Phosphate substrates, so that the binding of the phosphatase domain to DNA blocks the additional DNA binding of the kinase domain [39]. The priority activity of the phosphatase domain over the kinase domain may lie in the relative importance of the two activities against the most abundant type of DNA damage, which is 3'-phosphate termini [41].

It has been shown that disease-resistant domains and proteins are more able to tolerate variation, as they show significantly higher variant rates and less degree of sequence conservation than disease-susceptible proteins and domains [3]. In line with this, the site-directed mutagenesis of the kinase domain active site does not affect the phosphatase activity of PNKP. In contrast, the disruption of the phosphatase domain also abrogates kinase function when a 3'-phosphate in the substrate is found [39]. It was suggested that this inhibition of the kinase domain is the result of steric hindrance by the phosphatase domain substrate rather than by allosteric restructuring of the enzyme. Finally, it has been shown that the presence of a 3'-Phosphate completely blocks the repair of DNA breaks because it is not processable by either DNA polymerase or ligase. In contrast, a 5'-hydroxyl only blocks the ligation and this is suggested to be bypassed (somehow) and therefore be more tolerated [39].

Enzymes from both the SSBR/DSBR pathway have variants associated with pathological phenotypes like those associated with PNKP, but only variants in PNKP appear to combine both phenotypes (neurodevelopmental and neurodegeneration). The dual function of PNKP in DNA Damage Response allows the enzyme to participate in both SSBR and DSBR, but for reasons that remain unknown, the features that define which is the first affected pathway at the intracellular level in the nervous system is what determines the appearance of the clinical manifestations. Like germline deletion of other key components of base excision repair (BER), such as XRCC1, PNKP is essential for early embryogenesis [42]. Even when restricted to the nervous system, the deletion of PNKP still resulted in lethality [32]. Surprisingly, PNKP loss was substantially more severe than inactivation of either LIG4 (DSBR) or XRCC1 (SSBR) in mice, indicating the importance of this enzyme in repairing a broader range of DNA lesions than XRCC1 or LIG4 alone [32]. As neurons in the subventricular zone are reported to be very sensitive to the presence of unrepaired DSBs and readily undergo apoptosis, defective PNKP activity would lead to increased levels of apoptosis in the developing brain resulting in a reduced total brain volume as seen in MCSZ [43].

As none of the disease-causing mutations ablate the DNA 3’-phosphatase activity of PNKP, it is possible that MCSZ cells could retain residual levels of PNKP [31]. This residual activity can be sufficient to keep the levels of DNA damage below the threshold for apoptosis in post-mitotic neurons, which are not as sensitive as newly differentiated neurons in the developing brain [43]. In fact, as previously suggested by our group [44], it was recently demonstrated that the role PNKP is dependent on cellular stage and tissue. In a recent work, Shimada and colleagues irradiated fibroblasts, induced-Pluripotent Stem Cells (iPSCs) and Neural Progenitor Cells (NPCs) at 5 Gy. One hour after irradiation, RNA samples were extracted and analyzed by RNA-Seq. Interestingly, iPSCs but especially fibroblasts showed little dependence on PNKP to repair DSBs via NHEJ [45]. Instead, the NPCs showed a high level of PNKP transcription both at baseline and after irradiation. This could explain recent findings suggesting that DSBR is not the cause of the neuropathology associated with PNKP-mutated diseases. In this experiment, the authors only used patient-derived fibroblasts and were irradiated even at a lower dose (2 Gy) [46].

Another experiment using PNKP-deficient HCT116 and HeLa cells generated with CRISPR/Cas9 showed that cells were biochemically competent in removing both protruding and recessed 3'-phosphates from synthetic DSB substrates [47]. However, the removal was much less efficiently than WT cells, so the authors suggested an alternative 3'-phosphatase. In a recent work, only MCSZ cell lines exhibit a defect in repair of IR-induced SSBs implicating reduced PNKP-dependent DNA phosphatase [46]. The authors concluded that it is reduced DNA 5’-kinase activity that is the major contributor and/or cause of the neurodegeneration in PNKP-mutated disease. This may be true if we assume that the kinase domain is the main contributor to the disease because it is the only one that can generate a pathological (but still viable) phenotype.

**Fig 10:**
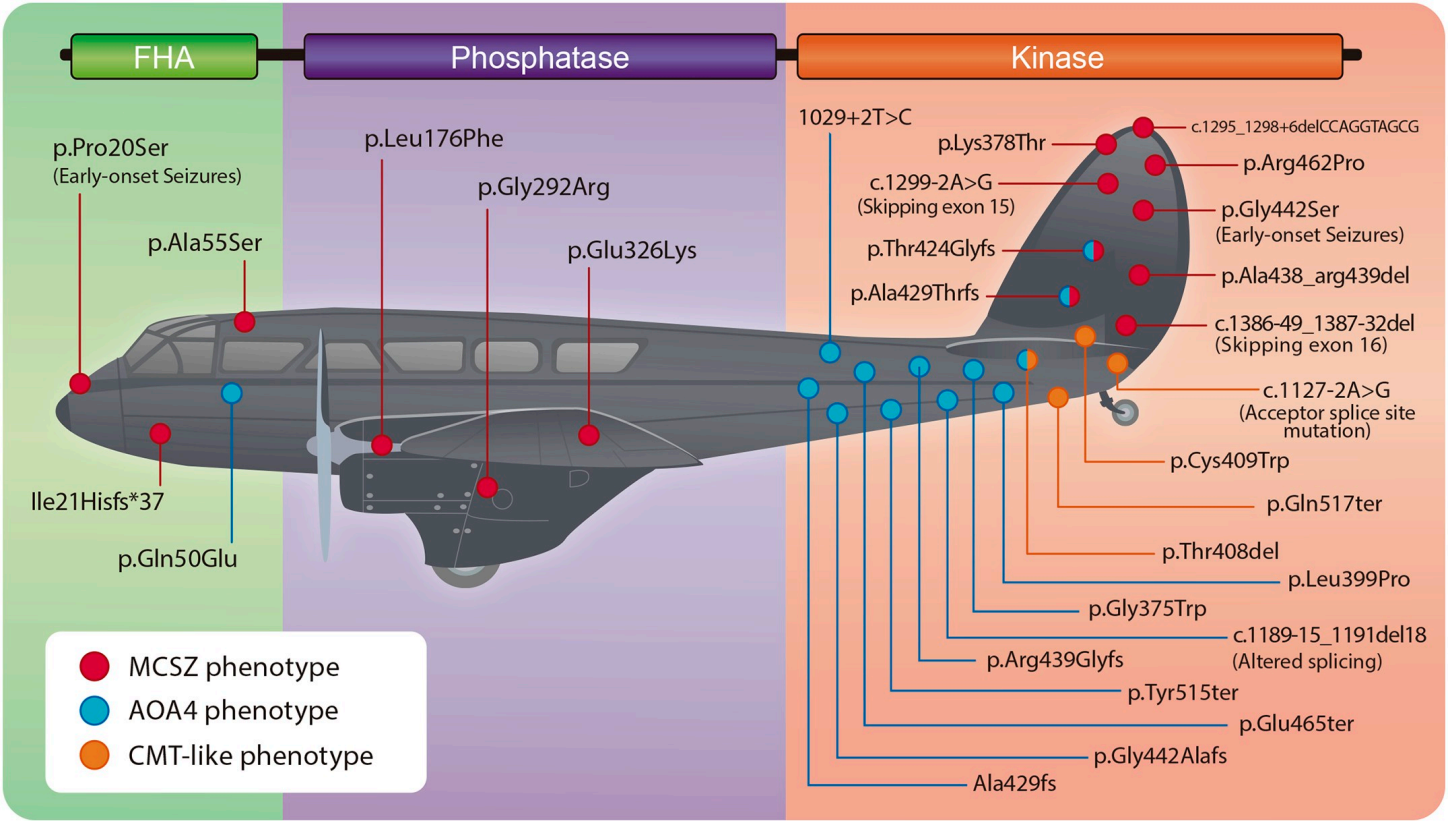
Depiction of PNKP as one of the airplanes from the Second World War from Wald’s analysis. The regions mutated within the kinase domain are the sites where the protein can be damaged and still generate a viable phenotype. In contrast, only three variants have been reported in the phosphatase domain and patients present the most severe phenotype: MCSZ. Therefore, the phosphatase domain represents the areas that when “attacked, would cause the plane to be lost”.

Even when all the variants in the kinase domain are deleterious, we found a greater variant tolerability for this domain due to higher dN/dS ratios. Accordingly, the kinase domain presented higher variant rates in both clinical reports and gnomAD dataset. It also showed lower degree of sequence conservation and fewer sites considered as disease-propensity hotspots compared to the phosphatase domain. This means that the kinase domain can tolerate serious damage in places such as the active site and still generate a viable phenotype. However, very few patients are reported with damage in the phosphatase domain, as most mutations in this domain are very unlikely to be viable. Since the majority of pathogenic variants observed in patients are in the kinase domain, we propose that variants in this domain represent “the areas where an airplane could suffer damage and still return" but the phosphatase domain, that is rarely mutated, represents the areas that “if attacked, would cause the plane to be lost” (Fig 10). This can be explained by the low variant rates, higher degree of sequence conservation, lower dN/dS ratios, and more disease-propensity hotspots. Nonetheless, more work should be done to fully demonstrate this survivorship bias at the experimental level.

Finally, we propose the term "Wald’s domain" for future studies analyzing the effect of variants in multidomain proteins and the possible survivorship bias. Based on our results, Wald’s domains would be characterized by lower O/E variants ratio, higher degree of sequence conservation, higher DPS and lower dN/dS ratios. Hence, our work may constitute a new approach to evaluate the effect of variants on protein structure and function.

## Conclusions

From Wald’s perspective, the regions mutated within the kinase domain are under survivorship bias since these sites represent points where the protein can be damaged and still generate a viable phenotype. It is also possible that factors other than the affected domain can influence the specific pathological phenotype observed (MCSZ, AOA, CMT). Taken together, our results support previous experimental evidence that demonstrates that the phosphatase domain is functionally more necessary and relevant for the repair of DNA insults, especially in the context of the development of the central nervous system. Our data illustrate the value and accuracy of available bioinformatics tools to determine the relationship between protein structure and function when analyzing the effect of multiple variants. We suggest considering survivorship bias when analyzing the role of variants in multidomain proteins.

## Methods

### Multiple sequence alignment

Human PNKP protein sequence and other seventy sequences from different species were collected, sixty-three obtained from Uniprot (http://www.uniprot.org) [48] and seven obtained from the NCBI Protein dataset (https://www.ncbi.nlm.nih.gov/protein). We chose the most representative species of each taxonomic group: 42 mammals, 8 bony fish, 5 reptiles, 3 amphibians, 3 birds, 2 arthropods, 2 apicomplexan, 1 amoebozoan, 1 cnidaria, 1 Mollusca, 1 bacterium and 1 virus (mimivirus). Uniprot: *Homo sapiens* (Q96T60), *Mus musculus* (Q9JLV6), *Rattus norvegicus* (A0A0G2JUH4), *Pan troglodytes* (K7BAU7), *Bos taurus* (F1N3Q9), *Anolis carolinensis* (G1KMT2), *Pongo abelii* (H2NZP9), *Ictidomys tridecemlineatus* (I3N446), *Otolemur garnettii* (H0WRT3), *Chlorocebus sabaeus* (A0A0D9S429), *Loxodonta africana* (G3TPU6), *Macaca fascicularis* (A0A2K5X601), *Saimiri boliviensis boliviensis* (A0A2K6TGX4), *Rhinopithecus bieti* (A0A2K6JZR0), *Colobus angolensis palliatus* (A0A2K5JFF3), *Propithecus coquereli* (A0A2K6FTG9), *Aotus nancymaae* (A0A2K5F2F6), *Cebus capucinus imitator* (A0A2K5R4X3), *Mesocricetus auratus* (A0A1U7R368), *Dipodomys ordii* (A0A1S3G4D4), *Mandrillus leucophaeus* (A0A2K5YE36), *Cavia porcellus* (H0V4R0), *Cricetulus griseus* (G3I715), *Myotis lucifugus* (G1PW59), *Ailuropoda melanoleuca* (G1M4Y9), *Capra hircus* (A0A452FU49), *Felis catus* (A0A337SAT2), *Sus scrofa* (F1RHU1), (*Ursus maritimus* (A0A452TNF4), *Ursus americanus* (A0A452QHV0), *Mustela putorius furo* (M3XT89), *Callithrix jacchus* (F6RVI4), *Drosophila melanogaster* (Q9VHS0), *Danio rerio* (Q08BP0), *Oryzias latipes* (H2MLN4), *Xenopus tropicalis* (B1WBK3), *Equus caballus* (F6QG90), *Xenopus laevis* (A9UMQ0), *Canis lupus familiaris* (E2R0U3), *Fopius arisanus* (A0A0C9R1P0), *Latimeria chalumnae* (H3B245), *Electrophorus electricus* (A0A4W4HJQ6), *Acanthamoeba castellanii* str. (L8GVC5), *Nothobranchius furzeri* (A0A1A7ZB78), *Alligator sinensis* (A0A1U7R7V0), *Austrofundulus limnaeus* (A0A2I4BRQ3), *Balaenoptera acutorostrata scammoni* (A0A384ACF1), *Ictalurus punctatus* (A0A2D0SWM3), *Gorilla gorilla gorilla* (G3R3W6), *Acanthamoeba polyphaga mimivirus* (Q5UQD2), *Nothobranchius kuhntae* (A0A1A8JP17), *Physeter macrocephalus* (A0A455AU26), *Alligator mississippiensis* (A0A151NJZ6), *Erinaceus europaeus* (A0A1S3AK18), *Hydra vulgaris* (T2M7D9), *Castor canadensis* (A0A250XY04), *Plasmodium malariae* (A0A1A8W4X2), *Neovison vison* (U6D1K9), *Callorhinus ursinus* (A0A3Q7NQ21), *Plasmodium gallinaceum* (A0A1J1GNL7), *Mycobacterium* sp. (A0A1A0X7E1), *Odobenus rosmarus divergens* (A0A2U3WM26), *Tarsius syrichta* (A0A1U7TJR7), *Vulpes vulpes* (A0A3Q7U9N3), *Delphinapterus leucas* (A0A2Y9P3J8). NCBI: *Gopherus evgoodei* (XP_030400898.1), *Microcaecilia unicolor* (XP_030053814.1), *Podarcis muralis* (XP_028557931.1), *Falco cherrug* (XP_027653441.1), *Lonchura striata domestica* (XP_021405459.1), *Gallus gallus* (XP_025001234.1), *Aplysia californica* (XP_005090552.1).

Multiple sequence alignment was performed with the EMBL-EBI’s server for Multiple Alignment using Fast Fourier Transform (MAFFT) [49] as it is recommended for better accuracy [50]. We also run a BlastP [23] analysis to obtain the percentage identity between the human PNKP sequence with respect to the rest of the species used in the alignment.

### Percentage identity of SSBR proteins

The human protein sequences of PNKP, PARP1, XRCC1, PARP1, and LIG3 were obtained from Uniprot [48] (Table 2). We also run a BlastP analysis to obtain the percentage identity of each human sequence with respect to seven different species (*Pan troglodytes*, *Equus caballus*, *Sus scrofa*, *Canis lupus*, *Felis catus*, *Mus musculus*, *Rattus norvegicus*, *Gallus gallus*, *Xenopus laevis*, *Danio rerio*, *Drosophila melanogaster*). The refseq_protein database (Max target sequences = 1000) was used and the sequences with the lowest E value, highest coverage and the highest similarity were chosen for each species. The difference in the sequence percentage identity was calculated and compared using the ANOVA test.

**Table 2:** Sequence ID used for the analysis of percent identity of each SSBR protein.

Species	POLB	PNKP	XRCC1	LIGIII	PARP1
*Pan troglodytes*	XP_009453547.1	XP_016792104.1	XP_016791569.1	XP_009430685.3	XP_003949754.2
*Equus caballus*	XP_001499852.3	XP_001917391.2	XP_001499917.3	XP_023508784.1	XP_023488446.1
*Sus scrofa*	XP_020933913.1	XP_005664806.3	XP_013844100.1	XP_020921813.1	XP_003357689.2
*Canis lupus*	XP_005629859.1	XP_005616345.1	NP_001273911.1	XP_005624869.1	XP_022277419.1
*Felis catus*	XP_003984799.1	XP_023101490.1	XP_003997757.1	XP_003996610.2	XP_003999138.3
*Mus musculus*	NP_035260.1	XP_006541101.2	NP_033558.3	NP_001278175.1	NP_031441.2
*Rattus norvegicus*	NP_058837.2	NP_001004259.1	XP_017445286.1	XP_008766242.1	NP_037195.1
*Gallus gallus*	XP_024998666.1	XP_025001234.1	XP_024997899.1	NP_001006215.2	NP_990594.2
*Xenopus laevis*	NP_001081643.1	NP_001108303.1	NP_001080711.1	NP_001082183.1	NP_001081571.1
*Danio rerio*	NP_001003879.2	NP_001071046.1	NP_001003988.2	NP_001025345.2	NP_001038407.1

### Conservation analysis

The Multiple Sequence Alignment previously generated was used as input in the ConSurf 2016 server [51] to estimate and visualize the conservation scores of the human PNKP protein sequence. The analysis was performed with default settings. The conservation scores for the amino acids within the phosphatase (146–337) and kinase (341–521) domain were analyzed and a homogeneity chi-squared test was performed to determine whether the conservation scores were different in each domain.

### Disease-propensity hotspots in PNKP

Based on both sequence and structure (Modeller) we analyzed the mutational sensitivity of the amino acids from the phosphatase and kinase domain using the SuSPect server [24]. This tool (Disease-Susceptibility-based SAV Phenotype Prediction) predicts how likely single amino acid variants (SAVs), are to be associated with the disease using a scale from 1–100. First, the average score of the 19 possible variants for each amino acid was calculated, and the distribution of said averages was plotted. We then plotted only the DPS greater than 50 to better visualize important differences. We performed the Kolmogorov–Smirnov test to verify the differences of both distributions. Then, we plotted the average DPS for the entire protein, defining the critical value as the 95th percentile, close to a DPS of 75. This means that, for a position to be considered as part of a “disease-propensity hotspot”, the region must present an average higher than 75 for all possible single amino acid variants.

### Human PNKP 3D structure prediction

As there is no crystal structure of the human PNKP protein in the RCSB Protein Data Bank (only the FHA domain based on the mouse’s structure) different servers were used to predict its 3D structure. We used Modeller [52], SWISS-MODEL [53], Phyre2 [54], and I-TASSER [55]. The modeling quality comparison was performed using the platform MolProbity [56], and the Structure Assessment tool of SWISS-MODEL. Final molecular graphics were elaborated using The PyMOL Molecular Graphics System, Version 2.0 Schrödinger, LLC (https://www.schrodinger.com/pymol). Structure alignment was performed with the TM-align server [57] using the structure from Modeller and the crystal structure of murine phosphatase-kinase domain (PDB ID: 3ZVL).

### PNKP ligand-binding sites prediction

We used the COACH [26] and COFACTOR [27] server to predict ligand-binding sites in PNKP. These predicted sites were compared to previously published experimental data [25, 28]. We also analyzed the potential post-translational modification (PTM) sites of PNKP by using NetPhos 3.1 [58]. To confirm these predictions, we reviewed the databases Phospho.ELM [59] and PhosphoSitePlus v6.5.9.1 [60] that contain *in vivo* and *in vitro* phosphorylation data.

### Structural damage of PNKP variants

We evaluated the structural damage of missense variants reported in PNKP using the 3D structure predicted with Modeller as input for the Missense3D server [61]. Dynamut [62] were also used to analyzed the impact of mutations on protein dynamics and stability resulting from vibrational entropy changes.

### PNKP variants in the general population and variant tolerability analysis

All reported (non-intronic) variants for PNKP were collected from both exome sequencing and whole genomes in the gnomAD v2.1.1 dataset [63] (https://gnomad.broadinstitute.org/). We only included variants from the canonical transcript (ENST00000322344.3). We especially analyzed “Missense” variants to compare the incidence between domains and analyze those cases reported as homozygous. Low confidence variants were not considered. To further obtain information about the mutational tolerability of PNKP, we used the MetaDome server [64] to plot the mutational tolerance landscape get the dN/dS ratios for each domain. Mann–Whitney U test was used to compare the average dN/dS ratio between domains and Kolmogorov–Smirnov test was used to verify the differences of both distributions.

## Supporting information

S1 Fig Structure assessment of the PNKP 3D models. A) Ramachandran plots of the three models generated on each platform. B) Comparison of the model with a non-redundant set of PDB structures. C) Local quality estimate according to the local similarity to the target. The comparison of each model with a non-redundant set of PDB structures showed that this model has the lowest value for the Qualitative Model Energy ANalysis (QMEAN) (-4.34). This indicates whether the QMEAN score of the model is comparable to what one would expect from experimental structures of similar size. The QMEAN for the model generated in Phyre2 was -7.06 and for I-TASSER was -10.21 (QMEAN Z-scores around zero indicate good agreement between the model structure and experimental structures of similar size). The “Local Quality” plot shows for each residue of the model (reported on the x-axis), the expected similarity to the native structure (y-axis).
(TIF)

S2 Fig Number of missense variants in PNKP (per domain) reported in gnomAD. Twelve mutations were found in homozygous condition in specific regions of the world.
(TIF)

S1 Table Evaluation of the 3D models predicted by different servers. (DOCX)

S1 Graphical Abstract (TIF)

## References

[1] Liu J, Rost B. CHOP proteins into structural domain-like fragments. Proteins. 2004;55: 678–688. 10.1002/prot.20095 15103630

[2] Bornberg-Bauer E, Beaussart F, Kummerfeld SK, Teichmann SA, Weiner J. The evolution of domain arrangements in proteins and interaction networks. CMLS, Cell Mol Life Sci. 2005;62: 435–445. 10.1007/s00018-004-4416-1 15719170 PMC11924419

[3] Yates CM, Sternberg MJE. Proteins and Domains Vary in Their Tolerance of Non-Synonymous Single Nucleotide Polymorphisms (nsSNPs). Journal of Molecular Biology. 2013;425: 1274–1286. 10.1016/j.jmb.2013.01.026 23357174

[4] Petukh M, Kucukkal TG, Alexov E. On Human Disease-Causing Amino Acid Variants: Statistical Study of Sequence and Structural Patterns. Human Mutation. 2015;36: 524–534. 10.1002/humu.22770 25689729 PMC4409542

[5] Kucukkal TG, Petukh M, Li L, Alexov E. Structural and physico-chemical effects of disease and non-disease nsSNPs on proteins. Current Opinion in Structural Biology. 2015;32: 18–24. 10.1016/j.sbi.2015.01.003 25658850 PMC4511717

[6] David A, Razali R, Wass MN, Sternberg MJE. Protein-protein interaction sites are hot spots for disease-associated nonsynonymous SNPs. Hum Mutat. 2012;33: 359–363. 10.1002/humu.21656 22072597

[7] David A, Sternberg MJE. The Contribution of Missense Mutations in Core and Rim Residues of Protein–Protein Interfaces to Human Disease. Journal of Molecular Biology. 2015;427: 2886–2898. 10.1016/j.jmb.2015.07.004 26173036 PMC4548493

[8] Gao M, Zhou H, Skolnick J. Insights into Disease-Associated Mutations in the Human Proteome through Protein Structural Analysis. Structure. 2015;23: 1362–1369. 10.1016/j.str.2015.03.028 26027735 PMC4497952

[9] Mangel M, Samaniego FJ. Abraham Wald’s Work on Aircraft Survivability. Journal of the American Statistical Association. 1984;79: 259–267. 10.1080/01621459.1984.10478038

[10] Op ‘t Eynde J, Yu AW, Eckersley CP, Bass CR. Primary blast wave protection in combat helmet design: A historical comparison between present day and World War I. Zonies D, editor. PLoS ONE. 2020;15: e0228802 10.1371/journal.pone.0228802 32053658 PMC7018002

[11] Stoltzfus A, Norris RW. On the Causes of Evolutionary Transition: Transversion Bias. Mol Biol Evol. 2016;33: 595–602. 10.1093/molbev/msv274 26609078 PMC7107541

[12] Gussow AB, Petrovski S, Wang Q, Allen AS, Goldstein DB. The intolerance to functional genetic variation of protein domains predicts the localization of pathogenic mutations within genes. Genome Biol. 2016;17: 9 10.1186/s13059-016-0869-4 26781712 PMC4717634

[13] Jilani A, Ramotar D, Slack C, Ong C, Yang XM, Scherer SW, et al Molecular Cloning of the Human Gene, *PNKP*, Encoding a Polynucleotide Kinase 3′-Phosphatase and Evidence for Its Role in Repair of DNA Strand Breaks Caused by Oxidative Damage. J Biol Chem. 1999;274: 24176–24186. 10.1074/jbc.274.34.24176 10446192

[14] Weinfeld M, Mani RS, Abdou I, Aceytuno RD, Glover JNM. Tidying up loose ends: the role of polynucleotide kinase/phosphatase in DNA strand break repair. Trends in Biochemical Sciences. 2011;36: 262–271. 10.1016/j.tibs.2011.01.006 21353781 PMC3134258

[15] Della-Maria J, Hegde ML, McNeill DR, Matsumoto Y, Tsai M-S, Ellenberger T, et al The Interaction between Polynucleotide Kinase Phosphatase and the DNA Repair Protein XRCC1 Is Critical for Repair of DNA Alkylation Damage and Stable Association at DNA Damage Sites. J Biol Chem. 2012;287: 39233–39244. 10.1074/jbc.M112.369975 22992732 PMC3493963

[16] Aceytuno RD, Piett CG, Havali-Shahriari Z, Edwards RA, Rey M, Ye R, et al Structural and functional characterization of the PNKP–XRCC4–LigIV DNA repair complex. Nucleic Acids Research. 2017;45: 6238–6251. 10.1093/nar/gkx275 28453785 PMC5449630

[17] Shen J, Gilmore EC, Marshall CA, Haddadin M, Reynolds JJ, Eyaid W, et al Mutations in PNKP cause microcephaly, seizures and defects in DNA repair. Nat Genet. 2010;42: 245–249. 10.1038/ng.526 20118933 PMC2835984

[18] Bras J, Alonso I, Barbot C, Costa MM, Darwent L, Orme T, et al Mutations in PNKP Cause Recessive Ataxia with Oculomotor Apraxia Type 4. The American Journal of Human Genetics. 2015;96: 474–479. 10.1016/j.ajhg.2015.01.005 25728773 PMC4375449

[19] Gatti M, Magri S, Nanetti L, Sarto E, Di Bella D, Salsano E, et al From congenital microcephaly to adult onset cerebellar ataxia: Distinct and overlapping phenotypes in patients with *PNKP* gene mutations. Am J Med Genet. 2019;179: 2277–2283. 10.1002/ajmg.a.61339 31436889

[20] Leal A, Bogantes-Ledezma S, Ekici AB, Uebe S, Thiel CT, Sticht H, et al The polynucleotide kinase 3′-phosphatase gene (PNKP) is involved in Charcot-Marie-Tooth disease (CMT2B2) previously related to MED25. Neurogenetics. 2018;19: 215–225. 10.1007/s10048-018-0555-7 30039206 PMC6280876

[21] Pedroso JL, Rocha CRR, Macedo-Souza LI, De Mario V, Marques W, Barsottini OGP, et al Mutation in *PNKP* presenting initially as axonal Charcot-Marie-Tooth disease. Neurol Genet. 2015;1: e30 10.1212/NXG.0000000000000030 27066567 PMC4811384

[22] Letunic I, Bork P. Interactive Tree Of Life (iTOL) v4: recent updates and new developments. Nucleic Acids Research. 2019;47: W256–W259. 10.1093/nar/gkz239 30931475 PMC6602468

[23] Altschul SF, Gish W, Miller W, Myers EW, Lipman DJ. Basic local alignment search tool. Journal of Molecular Biology. 1990;215: 403–410. 10.1016/S0022-2836(05)80360-2 2231712

[24] Yates CM, Filippis I, Kelley LA, Sternberg MJE. SuSPect: Enhanced Prediction of Single Amino Acid Variant (SAV) Phenotype Using Network Features. Journal of Molecular Biology. 2014;426: 2692–2701. 10.1016/j.jmb.2014.04.026 24810707 PMC4087249

[25] Garces F, Pearl LH, Oliver AW. The Structural Basis for Substrate Recognition by Mammalian Polynucleotide Kinase 3′ Phosphatase. Molecular Cell. 2011;44: 385–396. 10.1016/j.molcel.2011.08.036 22055185 PMC4820033

[26] Yang J, Roy A, Zhang Y. Protein–ligand binding site recognition using complementary binding-specific substructure comparison and sequence profile alignment. Bioinformatics. 2013;29: 2588–2595. 10.1093/bioinformatics/btt447 23975762 PMC3789548

[27] Zhang C, Freddolino PL, Zhang Y. COFACTOR: improved protein function prediction by combining structure, sequence and protein–protein interaction information. Nucleic Acids Research. 2017;45: W291–W299. 10.1093/nar/gkx366 28472402 PMC5793808

[28] Bernstein NK, Williams RS, Rakovszky ML, Cui D, Green R, Karimi-Busheri F, et al The Molecular Architecture of the Mammalian DNA Repair Enzyme, Polynucleotide Kinase. Molecular Cell. 2005;17: 657–670. 10.1016/j.molcel.2005.02.012 15749016

[29] Nakashima M, Takano K, Osaka H, Aida N, Tsurusaki Y, Miyake N, et al Causative novel PNKP mutations and concomitant PCDH15 mutations in a patient with microcephaly with early-onset seizures and developmental delay syndrome and hearing loss. J Hum Genet. 2014;59: 471–474. 10.1038/jhg.2014.51 24965255

[30] Chen MJ, Dixon JE, Manning G. Genomics and evolution of protein phosphatases. Sci Signal. 2017;10: eaag1796 10.1126/scisignal.aag1796 28400531

[31] Reynolds JJ, Walker AK, Gilmore EC, Walsh CA, Caldecott KW. Impact of PNKP mutations associated with microcephaly, seizures and developmental delay on enzyme activity and DNA strand break repair. Nucleic Acids Research. 2012;40: 6608–6619. 10.1093/nar/gks318 22508754 PMC3413127

[32] Shimada M, Dumitrache LC, Russell HR, McKinnon PJ. Polynucleotide kinase–phosphatase enables neurogenesis via multiple DNA repair pathways to maintain genome stability. EMBO J. 2015;34: 2465–2480. 10.15252/embj.201591363 26290337 PMC4601665

[33] Andressoo J-O, Jans J, de Wit J, Coin F, Hoogstraten D, van de Ven M, et al Rescue of Progeria in Trichothiodystrophy by Homozygous Lethal Xpd Alleles. Liu E, editor. PLoS Biol. 2006;4: e322 10.1371/journal.pbio.0040322 17020410 PMC1584416

[34] Campopiano R, Ferese R, Buttari F, Femiano C, Centonze D, Fornai F, et al A Novel Homozygous Variant in the Fork-Head-Associated Domain of Polynucleotide Kinase Phosphatase in a Patient Affected by Late-Onset Ataxia With Oculomotor Apraxia Type 4. Front Neurol. 2020;10: 1331 10.3389/fneur.2019.01331 32010037 PMC6974581

[35] Kalasova I, Hanzlikova H, Gupta N, Li Y, Altmüller J, Reynolds JJ, et al Novel PNKP mutations causing defective DNA strand break repair and PARP1 hyperactivity in MCSZ. Neurol Genet. 2019;5: e320 10.1212/NXG.0000000000000320 31041400 PMC6454307

[36] Veitia RA, Birchler JA. Dominance and gene dosage balance in health and disease: why levels matter!: Dominance and gene dosage balance in health and disease. J Pathol. 2010;220: 174–185. 10.1002/path.2623 19827001

[37] Veitia RA, Caburet S, Birchler JA. Mechanisms of Mendelian dominance: VEITIA et al. Clin Genet. 2018;93: 419–428. 28755412 10.1111/cge.13107

[38] Huber CD, Durvasula A, Hancock AM, Lohmueller KE. Gene expression drives the evolution of dominance. Nat Commun. 2018;9: 2750 10.1038/s41467-018-05281-7 30013096 PMC6048131

[39] Dobson CJ. The phosphatase activity of mammalian polynucleotide kinase takes precedence over its kinase activity in repair of single strand breaks. Nucleic Acids Research. 2006;34: 2230–2237. 10.1093/nar/gkl275 16648365 PMC1450335

[40] Breslin C, Caldecott KW. DNA 3’-Phosphatase Activity Is Critical for Rapid Global Rates of Single-Strand Break Repair following Oxidative Stress. Molecular and Cellular Biology. 2009;29: 4653–4662. 10.1128/MCB.00677-09 19546231 PMC2725712

[41] Ward JF. Nature of Lesions Formed by Ionizing Radiation In: Nickoloff JA, Hoekstra MF, editors. DNA Damage and Repair. Totowa, NJ: Humana Press; 1998 pp. 65–84. 10.1007/978-1-59259-455-9_5

[42] Tebbs RS, Flannery ML, Meneses JJ, Hartmann A, Tucker JD, Thompson LH, et al Requirement for theXrcc1DNA Base Excision Repair Gene during Early Mouse Development. Developmental Biology. 1999;208: 513–529. 10.1006/dbio.1999.9232 10191063

[43] Reynolds JJ, Stewart GS. A single strand that links multiple neuropathologies in human disease. Brain. 2013;136: 14–27. 10.1093/brain/aws310 23365091

[44] Bermúdez-Guzmán L, Leal A. DNA repair deficiency in neuropathogenesis: when all roads lead to mitochondria. Transl Neurodegener. 2019;8: 14 10.1186/s40035-019-0156-x 31110700 PMC6511134

[45] Shimada M, Tsukada K, Kagawa N, Matsumoto Y. Reprogramming and differentiation-dependent transcriptional alteration of DNA damage response and apoptosis genes in human induced pluripotent stem cells. Journal of Radiation Research. 2019; rrz057. 10.1093/jrr/rrz057 31665364 PMC7357234

[46] Kalasova I, Hailstone R, Bublitz J, Bogantes J, Hofmann W, Leal A, et al Pathological mutations in PNKP trigger defects in DNA single-strand break repair but not DNA double-strand break repair. Nucleic Acids Research. 2020; gkaa489. 10.1093/nar/gkaa489 32504494 PMC7337934

[47] Chalasani SL, Kawale AS, Akopiants K, Yu Y, Fanta M, Weinfeld M, et al Persistent 3′-phosphate termini and increased cytotoxicity of radiomimetic DNA double-strand breaks in cells lacking polynucleotide kinase/phosphatase despite presence of an alternative 3′-phosphatase. DNA Repair. 2018;68: 12–24. 10.1016/j.dnarep.2018.05.002 29807321 PMC6050096

[48] Apweiler R. UniProt: the Universal Protein knowledgebase. Nucleic Acids Research. 2004;32: 115D–119. 10.1093/nar/gkh131 14681372 PMC308865

[49] Madeira F, Park Y mi, Lee J, Buso N, Gur T, Madhusoodanan N, et al The EMBL-EBI search and sequence analysis tools APIs in 2019. Nucleic Acids Research. 2019;47: W636–W641. 10.1093/nar/gkz268 30976793 PMC6602479

[50] Pais FS-M, de Ruy P, Oliveira G, Coimbra R. Assessing the efficiency of multiple sequence alignment programs. Algorithms Mol Biol. 2014;9: 4 10.1186/1748-7188-9-4 24602402 PMC4015676

[51] Ashkenazy H, Abadi S, Martz E, Chay O, Mayrose I, Pupko T, et al ConSurf 2016: an improved methodology to estimate and visualize evolutionary conservation in macromolecules. Nucleic Acids Res. 2016;44: W344–W350. 10.1093/nar/gkw408 27166375 PMC4987940

[52] Webb B, Sali A. Comparative Protein Structure Modeling Using MODELLER. Current Protocols in Bioinformatics. 2016;54 10.1002/cpbi.3 27322406 PMC5031415

[53] Arnold K, Bordoli L, Kopp J, Schwede T. The SWISS-MODEL workspace: a web-based environment for protein structure homology modelling. Bioinformatics. 2006;22: 195–201. 10.1093/bioinformatics/bti770 16301204

[54] Kelley LA, Mezulis S, Yates CM, Wass MN, Sternberg MJE. The Phyre2 web portal for protein modeling, prediction and analysis. Nat Protoc. 2015;10: 845–858. 10.1038/nprot.2015.053 25950237 PMC5298202

[55] Roy A, Kucukural A, Zhang Y. I-TASSER: a unified platform for automated protein structure and function prediction. Nat Protoc. 2010;5: 725–738. 10.1038/nprot.2010.5 20360767 PMC2849174

[56] Williams CJ, Headd JJ, Moriarty NW, Prisant MG, Videau LL, Deis LN, et al MolProbity: More and better reference data for improved all-atom structure validation: PROTEIN SCIENCE.ORG. Protein Science. 2018;27: 293–315. 10.1002/pro.3330 29067766 PMC5734394

[57] Zhang Y. TM-align: a protein structure alignment algorithm based on the TM-score. Nucleic Acids Research. 2005;33: 2302–2309. 10.1093/nar/gki524 15849316 PMC1084323

[58] Blom N, Gammeltoft S, Brunak S. Sequence and structure-based prediction of eukaryotic protein phosphorylation sites. Journal of Molecular Biology. 1999;294: 1351–1362. 10.1006/jmbi.1999.3310 10600390

[59] Dinkel H, Chica C, Via A, Gould CM, Jensen LJ, Gibson TJ, et al Phospho.ELM: a database of phosphorylation sites—update 2011. Nucleic Acids Research. 2011;39: D261–D267. 10.1093/nar/gkq1104 21062810 PMC3013696

[60] Hornbeck PV, Zhang B, Murray B, Kornhauser JM, Latham V, Skrzypek E. PhosphoSitePlus, 2014: mutations, PTMs and recalibrations. Nucleic Acids Research. 2015;43: D512–D520. 10.1093/nar/gku1267 25514926 PMC4383998

[61] Ittisoponpisan S, Islam SA, Khanna T, Alhuzimi E, David A, Sternberg MJE. Can Predicted Protein 3D Structures Provide Reliable Insights into whether Missense Variants Are Disease Associated? Journal of Molecular Biology. 2019;431: 2197–2212. 10.1016/j.jmb.2019.04.009 30995449 PMC6544567

[62] Rodrigues CH, Pires DE, Ascher DB. DynaMut: predicting the impact of mutations on protein conformation, flexibility and stability. Nucleic Acids Research. 2018;46: W350–W355. 10.1093/nar/gky300 29718330 PMC6031064

[63] Karczewski KJ, Francioli LC, Tiao G, Cummings BB, Alföldi J, Wang Q, et al Variation across 141,456 human exomes and genomes reveals the spectrum of loss-of-function intolerance across human protein-coding genes. Genomics; 2019 1.

[64] Wiel L, Baakman C, Gilissen D, Veltman JA, Vriend G, Gilissen C. MetaDome: Pathogenicity analysis of genetic variants through aggregation of homologous human protein domains. Human Mutation. 2019; humu.23798. 10.1002/humu.23798 31116477 PMC6772141

