# Supplementary figures and images for: Mutational survivorship bias: The case of PNKP
Source: PLoS One. 2020 Dec 17;15(12):e0237682. doi: 10.1371/journal.pone.0237682 (PMC7746193; doi:10.1371/journal.pone.0237682)

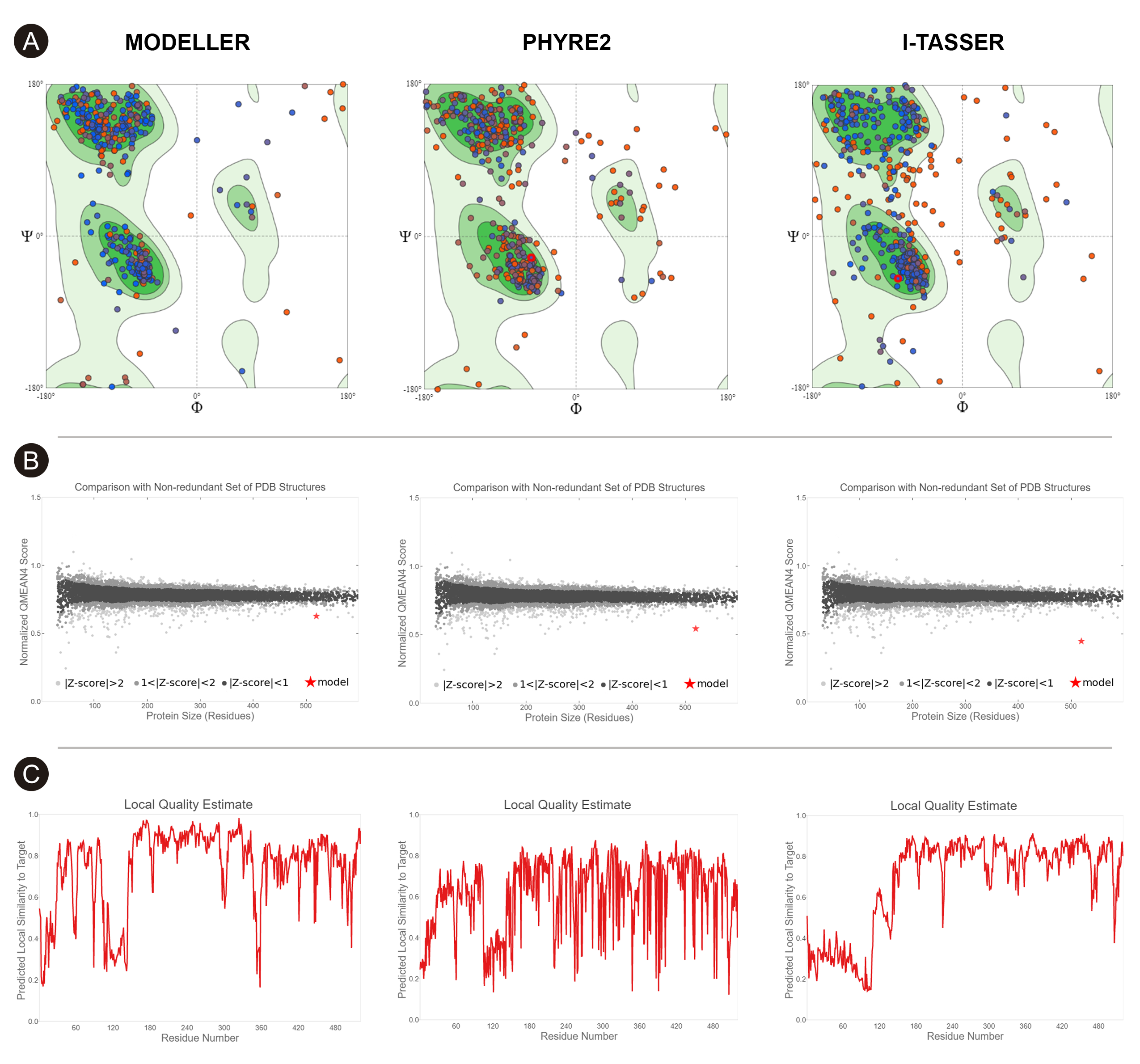

Supplement: S1 Fig — A) Ramachandran plots of the three models generated on each platform. B) Comparison of the model with a non-redundant set of PDB structures. C) Local quality estimate according to the local similarity to the target. The comparison of each model with a non-redundant set of PDB structures showed that this model has the lowest value for the Qualitative Model Energy ANalysis (QMEAN) (-4.34). This indicates whether the QMEAN score of the model is comparable to what one would expect from experimental structures of similar size. The QMEAN for the model generated in Phyre2 was -7.06 and for I-TASSER was -10.21 (QMEAN Z-scores around zero indicate good agreement between the model structure and experimental structures of similar size). The “Local Quality” plot shows for each residue of the model (reported on the x-axis), the expected similarity to the native structure (y-axis). (TIF) [file pone.0237682.s001.tif]

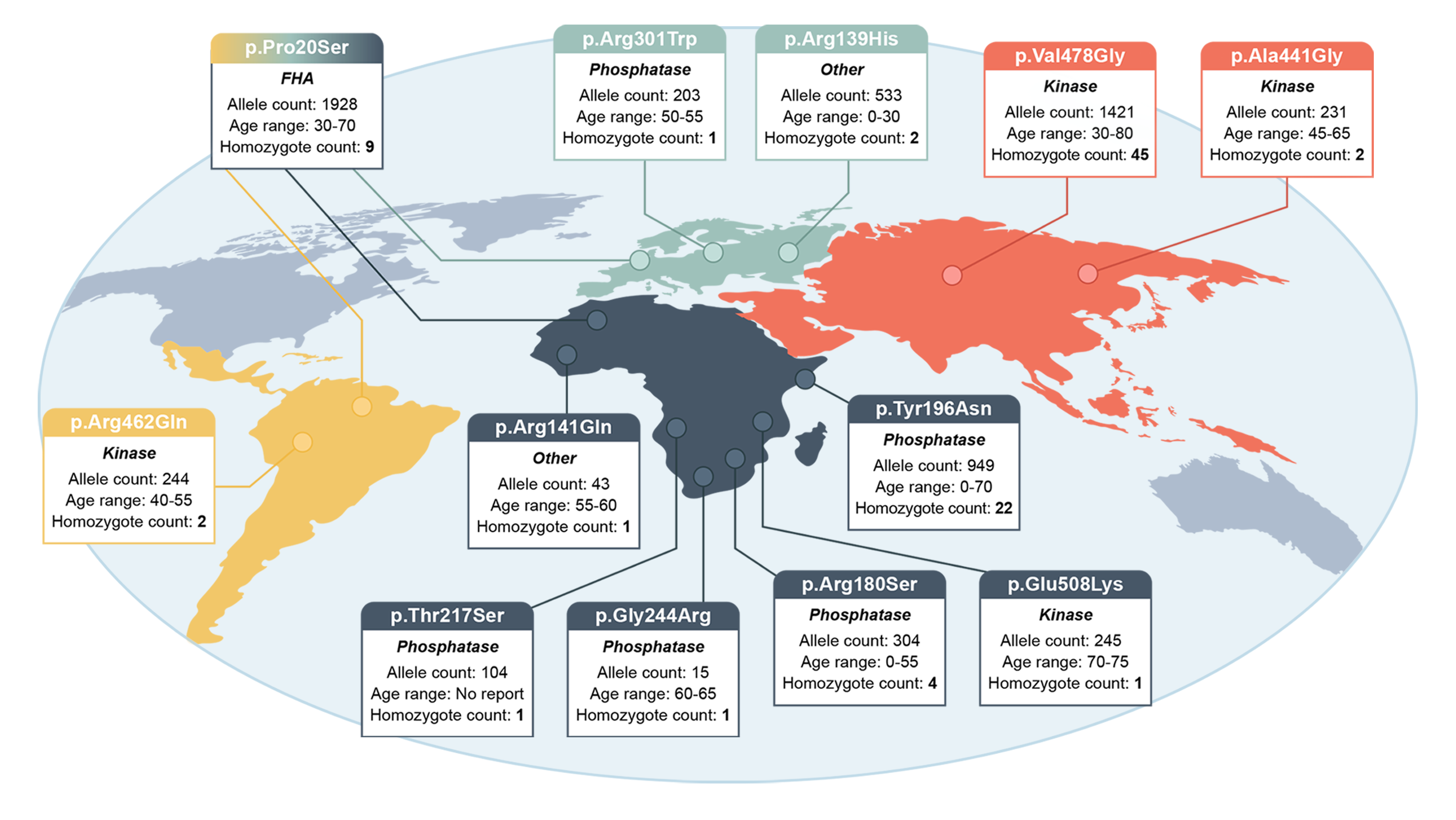

Supplement: S2 Fig — Twelve mutations were found in homozygous condition in specific regions of the world. (TIF) [file pone.0237682.s002.tif]

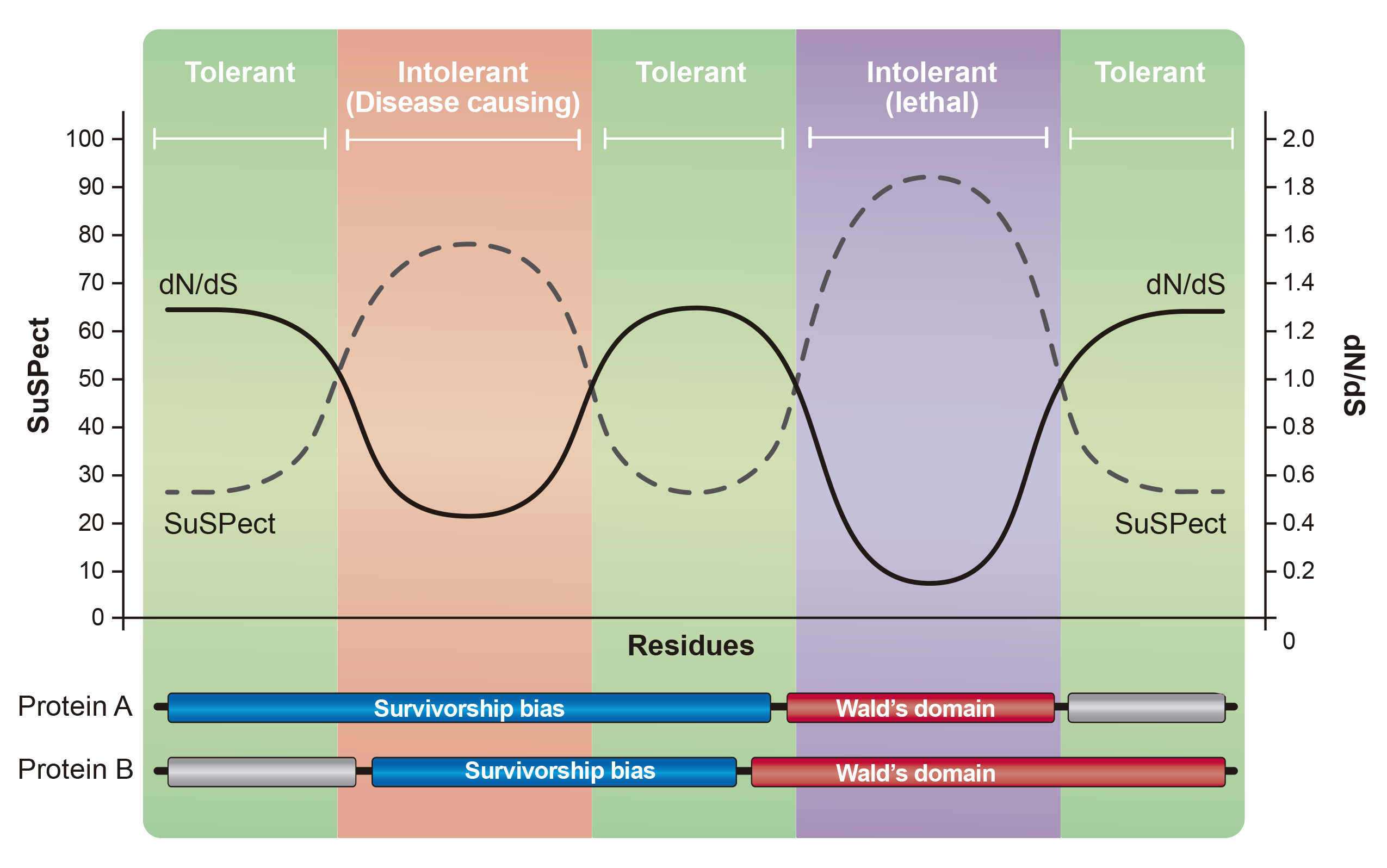

Supplement: S1 Graphical Abstract — (TIF) [file pone.0237682.s004.tif]
