# Supplementary material for: Mutational survivorship bias: The case of PNKP
Source: PLoS One. 2020 Dec 17;15(12):e0237682. doi: 10.1371/journal.pone.0237682 (PMC7746193; doi:10.1371/journal.pone.0237682)
Supplement: S1 Table — (DOCX) [file pone.0237682.s003.docx]

| **S1 Table. Evaluation of the 3D models predicted by different servers.Parameter** | | **Modeller** | | **PHYRE2** | | **I-TASSER** | | **Goal** |
| --- | --- | --- | --- | --- | --- | --- | --- | --- |
| Protein Geometry | Poor Rotamers | 32 | 7.44% | 25 | 5.81% | 80 | 18.60% | <0.3% |
|  | Favored rotamers | 370 | 86.05% | 383 | 89.07% | 305 | 70.93% | >98% |
|  | Ramachandran Outliers | 15 | 2.89% | 47 | 9.06% | 64 | 12.33% | <0.05% |
|  | Ramachandran favored | 484 | 93.26% | 413 | 79.58% | 380 | 73.22% | >98% |
|  | Cß deviations >0.25 Å | 58 | 12.24% | 227 | 47.89% | 76 | 16.03% | 0 |
|  | Bad bonds | 88/4124 | 2.03% | 227/4123 | 5.51% | 18/4123 | 0.44% | 0% |
|  | Bad angles | 301/5603 | 5.37% | 632/5603 | 11.28% | 285/5603 | 5.09% | <0.1% |
| Peptide Omegas | Cis prolines | 2/44 | 4.55% | 0/44 | 0.00% | 3/44 | 6.82% | <1 per chain or <5% |
|  | Twisted peptides | 3/520 | 0.58% | 2/520 | 0.38% | 78/520 | 15.00% | 0 |
| Low-resolution criteria | CaBLAM outliers | 20 | 3.9% | 40 | 7.7% | 77 | 14.90% | <1.0% |
|  | CA Geometry outliers | 11 | 2.13% | 11 | 2.13% | 58 | 11.22% | <0.5% |
| Additional validation | Tetrahedral geometry outliers | 24 | | 52 | | 21 | |  |
| MolProbity Score | | 3.80 | | 3.98 | | 3.99 | |  |
